# Genome-wide association study identifies variation at 6q25.1 associated with survival in multiple myeloma

**DOI:** 10.1038/ncomms10290

**Published:** 2016-01-08

**Authors:** David C. Johnson, Niels Weinhold, Jonathan S. Mitchell, Bowang Chen, Martin Kaiser, Dil B. Begum, Jens Hillengass, Uta Bertsch, Walter A. Gregory, David Cairns, Graham H. Jackson, Asta Försti, Jolanta Nickel, Per Hoffmann, Markus M. Nöethen, Owen W. Stephens, Bart Barlogie, Faith E. Davis, Kari Hemminki, Hartmut Goldschmidt, Richard S. Houlston, Gareth J. Morgan

**Affiliations:** 1 Division of Molecular Pathology, The Institute of Cancer Research, London SW7 3RP, UK; 2 Myeloma Institute, University of Arkansas for Medical Sciences, Little Rock, Arkansas 72205, USA; 3 Department of Internal Medicine V, University of Heidelberg, 69120 Heidelberg, Germany; 4 Division of Genetics and Epidemiology, The Institute of Cancer Research, London SW7 3RP, UK; 5 German Cancer Research Center, 69121 Heidelberg, Germany; 6 Leeds Institute of Molecular Medicine, Section of Clinical Trials Research, University of Leeds, Leeds LS2 9PH, UK; 7 Department of Haematology, Newcastle University, Newcastle-upon-Tyne NE1 7RU, UK; 8 Center for Primary Health Care Research, Lund University, 221 00 Malmö, Sweden; 9 Institute of Human Genetics, University of Bonn, D-53127 Bonn, Germany; 10 Division of Medical Genetics, Department of Biomedicine, University of Basel, 4031 Basel, Switzerland; 11 Department of Genomics, Life & Brain Center, University of Bonn, D-53127 Bonn, Germany; 12 National Center of Tumor Diseases, 69120 Heidelberg, Germany

## Abstract

Survival following a diagnosis of multiple myeloma (MM) varies between patients and some of these differences may be a consequence of inherited genetic variation. In this study, to identify genetic markers associated with MM overall survival (MM-OS), we conduct a meta-analysis of four patient series of European ancestry, totalling 3,256 patients with 1,200 MM-associated deaths. Each series is genotyped for ∼600,000 single nucleotide polymorphisms across the genome; genotypes for six million common variants are imputed using 1000 Genomes Project and UK10K as the reference. The association between genotype and OS is assessed by Cox proportional hazards model adjusting for age, sex, International staging system and treatment. We identify a locus at 6q25.1 marked by rs12374648 associated with MM-OS (hazard ratio=1.34, 95% confidence interval=1.22–1.48, *P*=4.69 × 10^–9^). Our findings have potential clinical implications since they demonstrate that inherited genotypes can provide prognostic information in addition to conventional tumor acquired prognostic factors.

## Abstract (web summary)

The prognosis of multiple myeloma patients varies widely. Here, to identify genetic factors associated with differing prognoses, the authors carried out a meta-analysis of four genome-wide association studies and identified a risk variant associated with survival interval.

## Graphical abstract

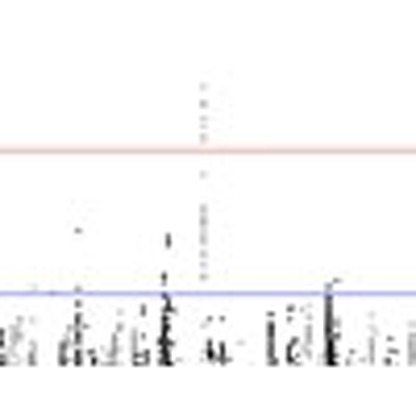

Multiple myeloma (MM) is a malignancy characterized by the infiltration of clonal plasma cells in the bone marrow^1^,^2^. There is considerable heterogeneity in the survival outcomes among MM patients and a variety of clinical features and tumour biomarkers have been shown to be predictive of prognosis^3^,^4^,^5^,^6^,^7^,^8^.

As a potential prognostic factor, the concept of germline variation imparting inter-individual variability in tumour development and progression is receiving increasing attention^9^,^10^,^11^,^12^. This observation is exemplified by breast cancer where a more accurate assessment of prognosis can be made by taking into account genetic information to improve therapeutic decision making, opening up the possibility of patient-tailored drug selection decisions^13^. In addition, detecting genes with prognostic relevance has the potential to aid the identification of pathways that could be targeted for novel therapeutic interventions.

Here to identify germline variation influencing patient outcome following a diagnosis of MM we have pooled genotype data from four independent genome-wide association studies (GWAS) of MM^14^,^15^,^16^,^17^ and linked these to survival time data. Our findings are consistent with the hypothesis that an individual's prognosis following treatment for MM is influenced by germline variation. Specifically, we identified a locus at 6q25.1 (rs12374648) associated with MM-OS that was statistically significant. Moreover, the association at 6q25.1 was consistently seen in each of the four patient cohorts and was not confined to a specific molecular subtype of MM.

## Results

### Genome-wide association study

**Figure 1:**
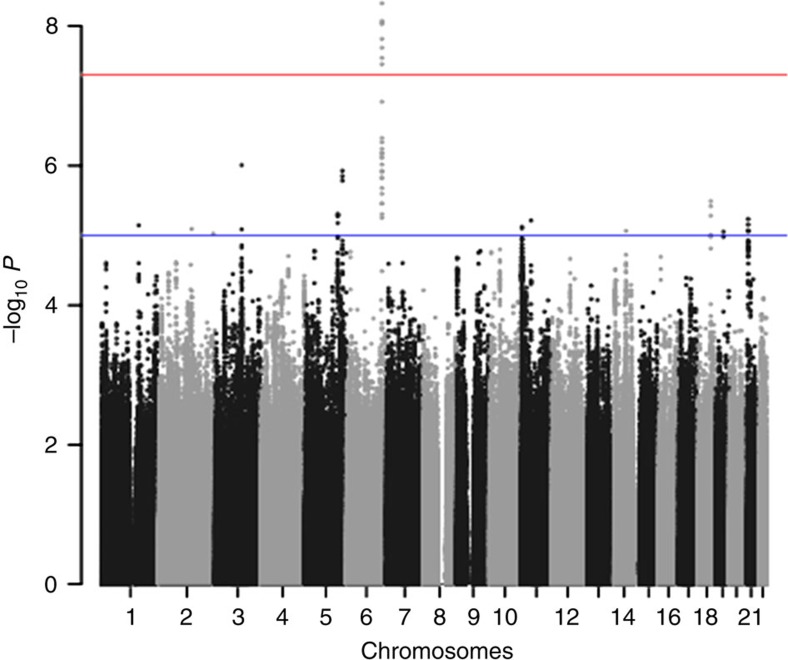
Association plot for combined analyses for MM-OS. The *P*-values of the association between each SNP and myeloma survival were obtained by Cox regression analyses with adjustment and then combined. The *y* axis shows the −log_10_ *P*-values of each SNP analysed, and the *x* axis shows their respective chromosome position. The red horizontal line corresponds to *P*=5.0 × 10^−8^. All statistical tests were two-sided.

**Figure 2:**
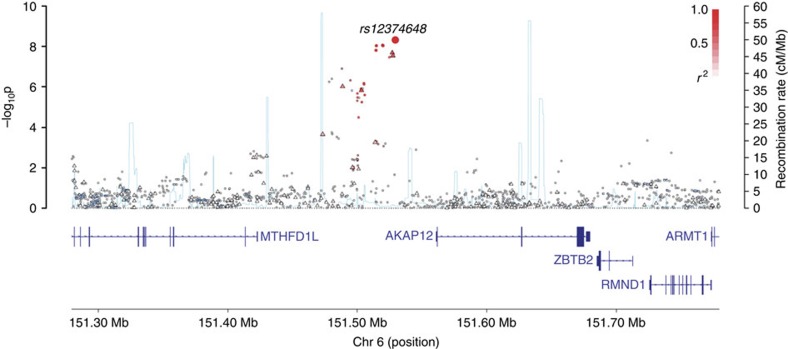
Regional plot of association results and recombination rates for the rs12374648 (6q25.1) MM-OS risk locus. Plots show association results of both genotyped (triangles) and imputed (circles) SNPs in the GWAS samples and recombination rates. −log_10_ *P*-values (*y* axes) of the SNPs are shown according to their chromosomal positions (*x* axes). The top genotyped SNP in each combined analysis is shown as a large diamond and is labelled by its rsID. The colour intensity of each symbol reflects the extent of LD with the top genotyped SNP, white (*r*^2^=0) through to dark red (*r*^2^=1.0). Genetic recombination rates, estimated using HapMap samples from Utah residents of western and northern European ancestry (CEU), are shown with a light blue line. Physical positions are based on NCBI build 37 of the human genome. Also shown are the relative positions of genes and transcripts mapping to the region of association. Genes have been redrawn to show their relative positions; therefore, maps are not to physical scale. Below each plot is a diagram of the exons and introns of the genes of interest.

**Figure 3:**
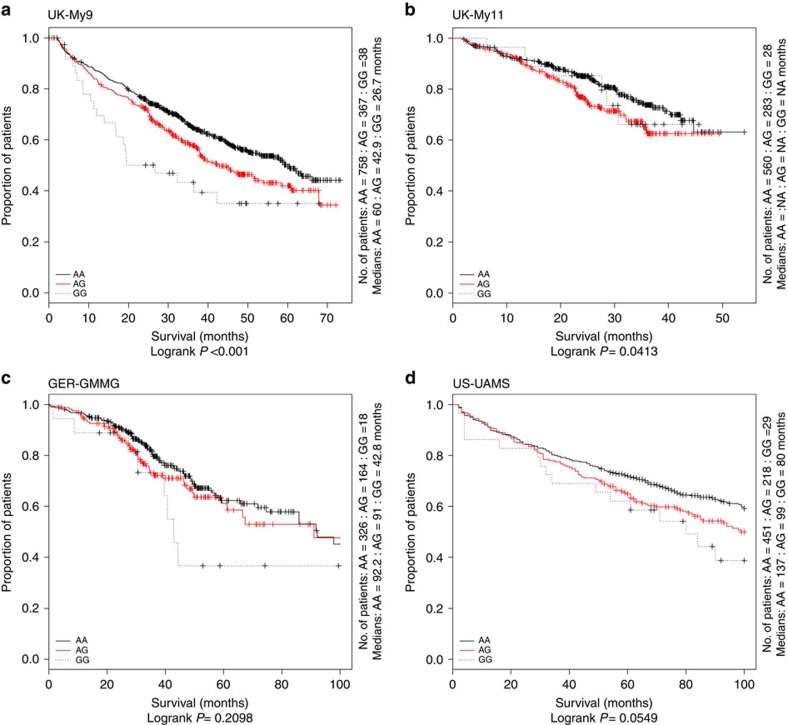
Kaplan–Meier curves for MM-OS at 6q25.1 (rs12374648). Survival curves for the AA homozygotes are shown as a solid line. The red line depicts the survival curve for the AG heterozygotes, and the dashed line depicts the survival curve for the rare homozygotes GG. Vertical ticks indicate censored data points.

After applying quality control measures, genotype data were available on 3,256 cases from the GWAS series, Supplementary Figs 1 and 2. The inflation factor four *λ* for each of the studies ranged from 0.99 to 1.03 and for the overall analysis was 1.02 (Supplementary Figs 3 and 4). We identified eight single nucleotide polymorphisms (SNPs) associated with MM-OS at *P* values ≤5.0 × 10^−8^; proportional hazards model. All eight SNPs were located on chromosome 6q25.1 and were in linkage disequilibrium (*r*^2^=0.58–1.0), Figs 1 and 2. The strongest association was provided by rs12748648 (*P*=4.89 × 10^−9^, hazard ratio (HR)=1.34, 95% confidence interval (CI)=1.22–1.48, risk allele frequency=0.19); Table 1, Fig. 2 and Supplementary Fig. 5. The association was consistent across the four series and there was very little between-study heterogeneity (*P*=0.34, *I*^2^=11%; test of heterogeneity). Homozygosity for rs12748648 GG was associated with median survival time of 26.7 (UK-My9), 42.8 (GER-German-speaking Myeloma Multicenter Study Group (GMMG)) and 80 (US-University of Arkansas for Medical Sciences (UAMS)) months as compared with 60, 92.2 and 137 months, respectively, for patients homozygous for AA genotype (Fig. 3). To address the possibility that the impact of rs12748648 on MM-OS is a consequence of its association with known cytogenetic risk factors we performed a multivariate analysis on the 1,165 patients of the UK-My9 cohort including rs12748648, high-risk IgH translocations, gain(1q21) and del(17p). This showed that 6p25.1 (rs12374648) independently impacted MM-OS (Supplementary Table 1). Patients with complete remission after autologous cell transplant (ASCT) tend to have a better survival. We examined whether the 6q25.1 association for OS was confined to patients with or without complete remission and we found the association was apparent in both patient groups (*P*=0.84; test of heterogeneity).

### Overall survival risk allele for myeloma at 6p25.1

rs12748648 maps within a 49.2-Kb (*r*^2^>0.2) region of linkage disequilibrium intergenic to *MTHFD1L* and *AKAP12* genes (Fig. 2). The genomic region contains multiple enhancer marks and the SNP localizes to a predicted enhancer element that is bound by TCF4 (TCF7L2), Supplementary Fig. 5. Analysis of eQTL data did not demonstrate a relationship between rs12748648 genotype and expression of *MTHFD1L*, *AKAP12* or distantly flanking genes (Supplementary Figs 6 and 7). Examining encyclopedia of DNA elements (ENCODE) CHIP-seq data in the lymphoblast cell line GM12878 of the 6p25.1 showed that rs12748648 maps to region enriched for H3K27me3, a polycomb repressive mark. As DNA methylation can have a role in access of such polycomb repression^18^, we undertook a meQTL analysis of the region around rs12748648. We found an association between rs12748648 risk genotype and reduced methylation of both *MTHFD1L* and *AKAP12* genes (*P*=0.0077 and 0.0097, respectively, log-linear regression; Supplementary Fig. 8). In addition to the 6q25.1 (rs12748648) association for MM-OS we identified suggestive associations (that is *P*<10^−5^; proportional hazards model) at 1q23.3 (rs1934908), 19q13.11 (rs1974807), 5q31.3 (rs2906053), 3q13 (rs4682170), 18q21 (rs57942319) and 2q22 (rs61070260; Supplementary Table 2 and Supplementary Figs 9–20). All of the SNPs defining these associations mapped to genomic regions with regulatory marks (Supplementary Table 3), with rs1934908 impacting *FCLRA* expression in plasma cells (Supplementary Fig. 21).

All of the SNP associations noted above for MM-OS showed a consistent effect on progression-free survival, Supplementary Table 4. It is possible that some of the inherited genetic variants that impact on the risk of developing MM^14^,^16^,^17^,^19^, may also impact on MM-OS. To address this possibility we examined the relationship between previously published risk SNPs and MM-OS. None of the nine validated risk SNPs for MM were associated with MM-OS, that is, *P*>0.05, proportional hazards model; Supplementary Table 5.

## Discussion

Our findings suggest a hypothesis that an individual's prognosis following treatment for MM is influenced by germline variation. In our analysis we adjusted for the influence of treatment on survival within studies, and report associations consistent across the four studies, thus our findings are likely to relate to underlying biology impacting the survival of the myeloma clone. The treatment used in all of the studies included ASCT, the GMMG studies used a tandem transplant, younger patients in the Medical Research Council (MRC) study received a single ASCT in both the Myeloma IX (UK-My9) and Myeloma IX (UK-My11) cases. The UAMS (US-UAMS) cases received predominantly tandem transplants together with combination induction and consolidation therapy. Prognostic factors generated from these studies are generally applicable to patients treated with ASCT and the use of novel agents including lenalidomide and bortezomib used as both induction and maintenance.

The 6q25.1 locus that associates with MM-OS spans a region of LD intergenic to *MTHFD1L* and *AKAP12* genes. While variation at 6q25.1 has previously been linked to coronary heart disease^20^,^21^ (rs6922269) and late-onset Alzheimer disease^22^ (rs11754661) the risk SNPs for these diseases are not correlated with rs12574648 (respective LD metrics–*r*^*2*^=0.002, *D*′=0.08 and *r*^*2*^=0.046, *D*′=0.65). *MTHFD1L* is involved in mitochondrial tetrahydrofolate (THF) synthesis^23^,^24^. One-carbon substituted forms of THF are important for the *de novo* synthesis of purines and thymidylate supporting cellular methylation by regenerating methionine from homocysteine. There have been no previous reports of associations of cancer risk or overall survival (OS) with variation at *AKAP12*, but *AKAP12* has been shown to be a tumour suppressor, acting through CyclinD1 (ref. 25). *AKAP12* is regulated by methylation in a number of cancers^26^,^27^,^28^,^29^,^30^,^31^,^32^,^33^ and is epigenetically repressed in MM, where its expression can be upregulated following treatment with DNA demethylation compounds such as trichostatin and/or 5-aza-2′-deoxycytidine^34^.

Intriguingly, although rare, 6q is a site of recurrent deletion in lymphoid tumours, that includes homozygous deletions at 6q25.3 (*AR1D1B/WTAP*)^35^ and mutations at 6q21 (*PRDM1/BLIMP1*)^36^,^37^,^38^. It is however unlikely that any somatic mutations in this regions are responsible for the MM-OS signal. While the functional basis for the rs12374648 remains to be established the SNP maps to a binding site for the transcription factor TCF7L2, alias TCF4. Binding of TCF7L2 correlates with hypomethylation and contributes to formation of differentially methylated regions of the genome^39^; TCF7L2 is commonly bound close to loci that are demethylated during differentiation^40^,^41^. Although speculative, we note that the rs12374648 risk genotype was associated with hypomethylation at the genomic region encompassing *MTHFD1L* and *AKAP12*, suggesting a possible mechanistic basis for the 6q25.1 association.

In addition to variation at 6q25.1, we identified suggestive associations at six other loci, a number of which annotate genes having strong *a priori* evidence for having a role in MM. Notably at 1q23.3, a region commonly amplified in MM, an association with rs1934908 genotype is also seen to be an eQTL for *FRCLA*, a gene with regulatory influence on IgG levels^42^. In contrast to these suggestive associations we did not find any evidence to support the recent claim that variation at 16p13 defined by the rare SNP rs72773978 influences the risk of MM-OS^43^, *P*_combined_=0.92, proportional hazards model; Supplementary Table 6.

GWASs have been successful in identifying variants influencing susceptibility for most cancers. Notably, nine common variants have thus far been shown to be associated with MM risk^14^,^15^,^16^,^17^,^19^. Paradoxically variants for cancer prognosis have been elusive and in this study we have only identified one variant for myeloma survival at genome-wide statistical significance. A major reason for the disparity is study power. Despite the size of our study, the power to detect association with MM-OS is at best only modest. All of the common susceptibility alleles for MM are associated with relative risks of ∼1.15 and such alleles can be identified through large case-control series, in contrast our survival analysis, based on 1,200 myeloma related deaths, had only 80% power to detect alleles with a HR greater ≥1.5 and minor allele frequency (MAF) >0.2.

Our findings support the hypothesis that germline variation influences outcome following treatment of MM. These results provide insight into the molecular mechanisms of tumour progression implying that germline markers of prognosis have the potential to enhance risk stratification. However, it is clear that trials larger than ours are required to identify additional loci associated with OS. Genotyping samples from future clinical trials, is likely to be especially informative and to offer the prospect of establishing the relationship between inherited variants, molecular subtypes and specific therapies.

## Methods

### Myeloma patient samples

We pooled data from four independent MM case series^14^,^15^,^16^,^17^ in populations of European ancestry with existing high-density SNP genotyping (Supplementary Fig. 1): (i) UK-My11-GWAS comprising 877 MM cases from the UK MRC Myeloma IX trial. (ii) UK-My9-GWAS comprising 1,165 MM cases from the UK MRC Myeloma IX trial; (iii) German-GMMG-GWAS, comprising 511 MM patients recruited by the GMMG, coordinated by the University Clinic, Heidelberg; (iv) US-UAMS-GWAS comprising 703 newly diagnosed MM patients treated at the Myeloma Institute at UAMS. All of these trials have been reported previously but importantly all include a single or double autologous stem cell transplant as one component of the treatment^44^,^45^,^46^,^47^ (Table 2). All studies were approved by the relevant institutional review boards, and all participants provided written informed consent.

### Genotyping and quality control

Cases were genotyped using Illumina Human OmniExpress-12 v1.0 arrays according to the manufacturer's protocols (Illumina, San Diego, USA). Standard quality control was performed on all scans, excluding individuals with low call rate (<90%) and extremely high or low heterozygosity (*P<*1.0 × 10^−4^, test of heterogeneity), as well as all individuals evaluated to be of non-European ancestry (using the HapMap version 2 CEU, JPT/CHB and YRI populations as a reference; Supplementary Fig. 2). A summary of the number of genotyped SNPs and the number of SNPs passing QC is detailed in Supplementary Fig. 1.

### Imputation

Genotypes for common variants across the genome were imputed using 1000 Genomes Project (phase 1 integrated release 3, March 2012) and UK10K as reference in conjunction with IMPUTE2 v2.1.1 (ref. 48) after pre-phasing with SHAPEIT software^49^. Poorly imputed SNPs defined by an information measure (Is)<0.90 were excluded from the analyses. All genomic locations are given in NCBI Build 37/UCSC hg19 coordinates.

### Statistical analysis

The primary end point was MM-specific OS (MM-OS). Time-to-event was calculated from date of recruitment (left censoring) to avoid bias from the inclusion of prevalent cases. In the German series, the follow-up was started from transplantation, shortly after diagnosis. Follow-up was right censored on the date of death if death was other than MM, or the date last known alive if death did not occur. Kaplan–Meier survival curves according to genotype were generated and the homogeneity of the survival curves between genotypes was examined. Cox regression analysis was used to estimate genotype-specific HR and associated 95% CI. Where necessary we controlled for cryptic population substructure by including a variable number of principal components as covariates for each data set. For each SNP HRs were generated using common allele homozygotes as the reference group. *P*-values presented correspond to the significance of a test difference among all three of the genotype groups (common allele homozygote, heterozygote and rare allele homozygote). For SNPs where fewer than five minor allele homozygotes were observed, minor allele homozygote genotypes were combined with the heterozygotes. For the statistically significantly associated SNPs, we ran multivariable Cox models adjusting for ISS, age and treatment where appropriate. We confined our analysis to SNPs with MAF >5% because of extreme value of the test statistics. Overall statistical significance tests for each SNP were performed by combining the results for each data set using a fixed-effects meta-analysis. All statistical tests were two-sided. Inflation of the test statistics, *λ*, was estimated by dividing the 45th percentile of the test statistic by 0.357—the 45th percentile for a *χ*^2^ distribution on 1 degree of freedom. Heterogeneity between studies was quantified using the *I*^2^ statistic. Associations were regarded as statistically significant at a *P* value ≤5.0 × 10^–8^ (that is, genome-wide significance). Multivariable stepwise variable selection was performed using a standard backward-elimination approach, variables were retained at a level of significance *P*<0.05. All statistical analyses were performed using R (v3.1.3) software^50^,^51^.

### eQTL analysis

Expression quantitative trait locus (eQTL) analyses were performed for all genes in the 1 MB region spanning the MM-OS associated SNPs using Affymetrix Human Genome U133+2.0 array data for plasma cells from 184 MRC Myeloma IX trial patients, 658 GMMG patients and 604 UAMS patients, analysis as previously described^52^. In addition, we surveyed the effect of genotype on expression in other tissues using the publicly accessible GTeX (ref. 53), Blood eQTL (ref. 54), MuTHER studies^55^, Framingham heart study^56^ and SCAN (ref. 57) databases.

### meQTL analysis

Methylation quantitative trait locus analyses of adjacent genes to SNPs of interest using probe-level DNA methylation data generated using Illumina 450 K methylation arrays on plasma cells from 365 MRC myeloma XI trial patients (UK-My11). Association between SNP genotype and normalized methylation levels was tested by linear regression.

### Translocation detection

Conventional cytogenetic studies of MM cells were conducted using standard karyotyping methodologies, and standard criteria for the definition of a clone were applied. Fluorescence *in situ* hybridization and ploidy classification of UK samples was conducted using methodologies previously described^58^,^59^. Fluorescence *in situ* hybridization and ploidy classification of GMMG samples was performed as previously described. The XL IGH Break Apart probe (MetaSystems, Altlussheim Germany) was used to detect any IGH translocation in GMMG^60^.

### Bioinformatics

To explore the epigenetic profile of association signals, we used chromatin state segmentation in lymphoblastoid cell lines (LCL) data generated by the ENCODE project. The states were inferred from ENCODE histone modification data (histone H4 Lys20 methylation (H4K20me1), H3 Lys9 acetylation (H3K9ac), H3K4me3, H3K4me2, H3K4me1, H3K36me3, H3K27me3, H3K27ac and CCCTC-binding factor (CTCF)) binarized using a multivariate hidden Markov model. We used HaploReg and RegulomeDB^61^,^62^ to examine whether any of the SNPs or their proxies (that is, *r*^2^>0.8 in the 1000 Genomes EUR reference panel) annotating putative transcription factor binding or enhancer elements. We assessed sequence conservation using Genomic Evolutionary Rate Profiling scores^63^.

## Additional information

**How to cite this article:** Johnson, D. C. *et al*. Genome-wide association study identifies variation at 6q25.1 associated with survival in multiple myeloma. *Nat. Commun.* 7:10290 doi: 10.1038/ncomms10290 (2016).

## Supplementary Material

Supplementary Information Supplementary Figures 1-21, Supplementary Tables 1-6 and Supplementary References.

## Figures and Tables

**Table 1:** Summary result for the combined analysis of 6q25.1 (rs12374648) and MM-OS.

**SNP**	**Base pair**	**Risk allele**	**Study**	**RAF**	**N**	**Events**	**Genoytypes**			**HR (95% CI)**	***P*****-value**
rs12374648 (6q25.1)	151529369	G					GG	AG	AA		
			UK-My9	0.19	1,163	510	38	367	758	1.45 (1.24–1.68)	1.69 × 10^−6^
			UK-My11	0.19	871	201	28	283	560	1.39 (1.10–1.75)	0.006
			GER-GMMG	0.20	508	156	18	164	326	1.09 (0.82–1.44)	0.55
			US-UAMS	0.20	702	330	30	218	454	1.28 (1.06–1.54)	0.009
			Combined							1.34 (1.22–1.48)	4.69 × 10^−9^*P*_het_=0.34, *I*^2^=11%

RAF, risk allele frequency.

The *P*-values of the association between each SNP and myeloma survival were obtained by Cox regression analyses with adjustment and then combined.

**Table 2:** Clinical characteristics in the four patient cohorts.

	**UK-My9**	**UK-My11**	**GER-GMMG**	**US-UAMS**
Number of cases	1,165	877	511	703
Median age at diagnosis	64	66	57	59
Median follow-up (months)	46.7	29.8	45.2	114
	48.0 (ASCT)	29.8 (ASCT)		
				
*Gender*				
Male	694	520	294	444
Female	471	357	217	259
				
*ISS*				
I	246	231	232	332
II	475	373	168	208
III	444	273	111	163
				
*WHO performance stage*				
0	302	316	177	NA
1	529	360	147	NA
2	210	139	24	NA
≥3	119	46	6	NA
NA	5	16	157	NA
				
*Deceased*				
Yes	653	201	156	335
No	512	676	355	368
				
*Disease progression*				
Yes	900	465	358	252
No	265	412	153	451
Autologous cell transplant	504	516	511	703
Study	Intensive: 504	Intensive: 516	HD3: 81	TT2: 248
	Non-intensive: 661	Non-intensive: 361	HD4: 275	TT3: 183
			Non-trial: 155	TT3B: 104
				TT4: 95
				Non-trial: 73
Treatment received	CTD: 358	CTD: 265	PAD: 175	DPACE-VAD: 128
	CTDa: 215	CTDa: 184	TAD: 52	DTPACE: 151
	CVAD: 346	CRD: 250	VAD: 263	MVTDPACE: 96
	MP: 237	CRDa: 178	Other: 21	VDTPACE: 11
	Other: 9			VDTPACEa: 182
				Other: 31
				
*Heavy chain paraprotein*				
IgG	635	464	297	393
IgA	226	221	116	150
IgD	23	17	4	5
LCO	134	112	82	129
None	8	6	7	11
NA/other	139	57	5	15
				
*Light chain paraprotein*				
Lambda	354	260	165	417
Kappa	672	560	340	258
No light chain	0	5	0	6
NA/other	139	52	6	22
				
*t4;14*				
Yes	72	81	49	NA
No	593	504	370	NA
NA	500	292	92	NA
				
*Gain 1q21*				
Yes	206	161	152	NA
No	360	297	245	NA
NA	599	419	114	NA
				
*del p53*				
Yes	60	41	37	NA
No	583	417	366	NA
NA	522	419	108	NA

CRD, cyclophosphamide, lenalidomide, dexamethasone; CTD, cyclophosphamide, thalidomide, dexamethasone; CVAD, cyclophosphamide, vincristine, doxorubicin, dexamethasone; DPACE, dexamethasone, cisplatin, doxorubicin, cyclophosphamide and etoposide; DTPACE, examethasone, thalidomide, cisplatin, doxorubicin, cyclophosphamide and etoposide; MP, melphalan, prednisone; MVDTPACE, melphalan, bortezomib, thalidomide, dexamethasone, cisplatin, doxorubicin, cyclophosphamide, etoposide; NA, not applicable; PAD, bortezomib, adriamycin, dexamethasone; TAD, thalidomide, doxorubicin, dexamethasone; TT, total therapy; VAD, vincristine, doxorubicin and dexamethasone; VDTPACE, bortezomib, thalidomide, dexamethasone, cisplatin, doxorubicin, cyclophosphamide, etoposide.

## References

[b1] Palumbo A. & Anderson K. Multiple myeloma. N. Engl. J. Med. 364, 1046–1060 (2011). 21410373 10.1056/NEJMra1011442

[b2] Kyle R. A. & Rajkumar S. V. Multiple myeloma. N. Engl. J. Med. 351, 1860–1873 (2004). 15509819 10.1056/NEJMra041875

[b3] Bergsagel P. L., Mateos M. V., Gutierrez N. C., Rajkumar S. V. & San Miguel J. F. Improving overall survival and overcoming adverse prognosis in the treatment of cytogenetically high-risk multiple myeloma. Blood 121, 884–892 (2013). 23165477 10.1182/blood-2012-05-432203 PMC3567336

[b4] Blade J., Rosinol L. & Cibeira M. T. Prognostic factors for multiple myeloma in the era of novel agents. Ann. Oncol. 19, vii117–vii120 (2008). 18790932 10.1093/annonc/mdn437

[b5] Tricot G. Prognostic factors in multiple myeloma. Clin. Adv. Hematol. Oncol. 3, 167–168 (2005). 16166987

[b6] Bergsagel P. L. Prognostic factors in multiple myeloma: it's in the genes. Clin. Cancer Res. 9, 533–534 (2003). 12576415

[b7] Rajkumar S. V. & Greipp P. R. Prognostic factors in multiple myeloma. Hematol. Oncol. Clin. North Am. 13, 1295–1314 (1999). 10626152 10.1016/s0889-8588(05)70128-3

[b8] Boyd K. D. . A novel prognostic model in myeloma based on co-segregating adverse FISH lesions and the ISS: analysis of patients treated in the MRC Myeloma IX trial. Leukemia 26, 349–355 (2012). 21836613 10.1038/leu.2011.204 PMC4545515

[b9] Lee J. C. . Human SNP links differential outcomes in inflammatory and infectious disease to a FOXO3-regulated pathway. Cell 155, 57–69 (2013). 24035192 10.1016/j.cell.2013.08.034 PMC3790457

[b10] Wu C. . Genome-wide association study identifies common variants in SLC39A6 associated with length of survival in esophageal squamous-cell carcinoma. Nat. Genet. 45, 632–638 (2013). 23644492 10.1038/ng.2638

[b11] Van Rechem C. . A coding single-nucleotide polymorphism in lysine demethylase KDM4A associates with increased sensitivity to mTOR inhibitors. Cancer Discov. 5, 245–254 (2015). 25564517 10.1158/2159-8290.CD-14-1159 PMC4355226

[b12] Eslam M. . Interferon-lambda rs12979860 genotype and liver fibrosis in viral and non-viral chronic liver disease. Nat. Commun. 6, 6422 (2015). 25740255 10.1038/ncomms7422 PMC4366528

[b13] Fasching P. A. . The role of genetic breast cancer susceptibility variants as prognostic factors. Hum. Mol. Genet. 21, 3926–3939 (2012). 22532573 10.1093/hmg/dds159 PMC3412377

[b14] Chubb D. . Common variation at 3q26.2, 6p21.33, 17p11.2 and 22q13.1 influences multiple myeloma risk. Nat. Genet. 45, 1221–1225 (2013). 23955597 10.1038/ng.2733 PMC5053356

[b15] Weinhold N. . The CCND1 c.870G>A polymorphism is a risk factor for t(11;14)(q13;q32) multiple myeloma. Nat. Genet. 45, 522–525 (2013). 23502783 10.1038/ng.2583 PMC5056630

[b16] Broderick P. . Common variation at 3p22.1 and 7p15.3 influences multiple myeloma risk. Nat. Genet. 44, 58–61 (2012). 22120009 10.1038/ng.993 PMC5108406

[b17] Erickson S. W. . Genome-wide scan identifies variant in 2q12.3 associated with risk for multiple myeloma. Blood 124, 2001–2003 (2014). 25237182 10.1182/blood-2014-07-586701

[b18] Reddington J. P. . Redistribution of H3K27me3 upon DNA hypomethylation results in de-repression of Polycomb target genes. Genome Biol. 14, R25 (2013). 23531360 10.1186/gb-2013-14-3-r25 PMC4053768

[b19] Swaminathan B. . Variants in ELL2 influencing immunoglobulin levels associate with multiple myeloma. Nat. Commun. 6, 7213 (2015). 26007630 10.1038/ncomms8213 PMC4455110

[b20] Hubacek J. A. . Rs6922269 marker at the MTHFD1L gene predict cardiovascular mortality in males after acute coronary syndrome. Mol. Biol. Rep. 42, 1289–1293 (2015). 25809277 10.1007/s11033-015-3870-1

[b21] Palmer B. R. . Genetic polymorphism rs6922269 in the MTHFD1L gene is associated with survival and baseline active vitamin B12 levels in post-acute coronary syndromes patients. PLoS ONE 9, e89029 (2014). 24618918 10.1371/journal.pone.0089029 PMC3949666

[b22] Naj A. C. . Dementia revealed: novel chromosome 6 locus for late-onset Alzheimer disease provides genetic evidence for folate-pathway abnormalities. PLoS Genet. 6, e1001130 (2010). 20885792 10.1371/journal.pgen.1001130 PMC2944795

[b23] Puissant A. . Targeting MYCN in neuroblastoma by BET bromodomain inhibition. Cancer Discov. 3, 308–323 (2013). 23430699 10.1158/2159-8290.CD-12-0418 PMC3672953

[b24] Delmore J. E. . BET bromodomain inhibition as a therapeutic strategy to target c-Myc. Cell 146, 904–917 (2011). 21889194 10.1016/j.cell.2011.08.017 PMC3187920

[b25] Lin X., Nelson P. & Gelman I. H. SSeCKS, a major protein kinase C substrate with tumor suppressor activity, regulates G(1)-->S progression by controlling the expression and cellular compartmentalization of cyclin D. Mol. Cell. Biol. 20, 7259–7272 (2000). 10982843 10.1128/mcb.20.19.7259-7272.2000 PMC86280

[b26] Liu W. . Quantitative assessment of AKAP12 promoter methylation in human prostate cancer using methylation-sensitive high-resolution melting: correlation with Gleason score. Urology 77, 1006 e1–1006 e7 (2011). 21310466 10.1016/j.urology.2010.12.010

[b27] Wu W., Zhang J., Yang H., Shao Y. & Yu B. Examination of AKAP12 promoter methylation in skin cancer using methylation-sensitive high-resolution melting analysis. Clin. Exp. Dermatol. 36, 381–385 (2011). 21198787 10.1111/j.1365-2230.2010.03968.x

[b28] Jo U. H., Whang Y. M., Sung J. S. & Kim Y. H. Methylation of AKAP12{alpha} promoter in lung cancer. Anticancer Res. 30, 4595–4600 (2010). 21115911

[b29] Mardin W. A. . SERPINB5 and AKAP12 - expression and promoter methylation of metastasis suppressor genes in pancreatic ductal adenocarcinoma. BMC Cancer 10, 549 (2010). 20939879 10.1186/1471-2407-10-549 PMC2966466

[b30] Liu W. . Quantitative assessment of AKAP12 promoter methylation in colorectal cancer using methylation-sensitive high resolution melting: Correlation with Duke's stage. Cancer Biol. Ther. 9, 862–871 (2010). 20364105 10.4161/cbt.9.11.11633

[b31] Liu W. . Rapid determination of AKAP12 promoter methylation levels in peripheral blood using methylation-sensitive high resolution melting (MS-HRM) analysis: application in colorectal cancer. Clin. Chim. Acta 411, 940–946 (2010). 20227403 10.1016/j.cca.2010.03.003

[b32] Flotho C., Paulun A., Batz C. & Niemeyer C. M. AKAP12, a gene with tumour suppressor properties, is a target of promoter DNA methylation in childhood myeloid malignancies. Br. J. Haematol. 138, 644–650 (2007). 17686059 10.1111/j.1365-2141.2007.06709.x

[b33] Turtoi A. . The angiogenesis suppressor gene AKAP12 is under the epigenetic control of HDAC7 in endothelial cells. Angiogenesis 15, 543–554 (2012). 22584896 10.1007/s10456-012-9279-8

[b34] Heller G. . Genome-wide transcriptional response to 5-aza-2'-deoxycytidine and trichostatin a in multiple myeloma cells. Cancer Res. 68, 44–54 (2008). 18172295 10.1158/0008-5472.CAN-07-2531

[b35] Walker B. A. . A compendium of myeloma-associated chromosomal copy number abnormalities and their prognostic value. Blood 116, e56–e65 (2010). 20616218 10.1182/blood-2010-04-279596

[b36] Chapman M. A. . Initial genome sequencing and analysis of multiple myeloma. Nature 471, 467–472 (2011). 21430775 10.1038/nature09837 PMC3560292

[b37] Lohr J. G. . Widespread genetic heterogeneity in multiple myeloma: implications for targeted therapy. Cancer Cell 25, 91–101 (2014). 24434212 10.1016/j.ccr.2013.12.015 PMC4241387

[b38] Walker B. A. . Mutational spectrum, copy number changes, and outcome: results of a sequencing study of patients with newly diagnosed myeloma. J. Clin. Oncol. 33, 3911–3920 (2015). 26282654 10.1200/JCO.2014.59.1503 PMC6485456

[b39] Kaaij L. T. . DNA methylation dynamics during intestinal stem cell differentiation reveals enhancers driving gene expression in the villus. Genome Biol. 14, R50 (2013). 23714178 10.1186/gb-2013-14-5-r50 PMC4053812

[b40] Bock C. . DNA methylation dynamics during *in vivo* differentiation of blood and skin stem cells. Mol. Cell 47, 633–647 (2012). 22841485 10.1016/j.molcel.2012.06.019 PMC3428428

[b41] Lewis A. . A polymorphic enhancer near GREM1 influences bowel cancer risk through differential CDX2 and TCF7L2 binding. Cell Rep. 8, 983–990 (2014). 25131200 10.1016/j.celrep.2014.07.020 PMC4471812

[b42] Wilson T. J., Gilfillan S. & Colonna M. Fc receptor-like A associates with intracellular IgG and IgM but is dispensable for antigen-specific immune responses. J. Immunol. 185, 2960–2967 (2010). 20668221 10.4049/jimmunol.1001428

[b43] Ziv E. . Genome-wide association study identifies variants at 16p13 associated with survival in multiple myeloma patients. Nat. Commun. 6, 7539 (2015). 26198393 10.1038/ncomms8539 PMC4656791

[b44] Morgan G. J. . Long-term follow-up of MRC Myeloma IX trial: Survival outcomes with bisphosphonate and thalidomide treatment. Clin. Cancer Res. 19, 6030–6038 (2013). 23995858 10.1158/1078-0432.CCR-12-3211

[b45] Morgan G. J. . Cyclophosphamide, thalidomide, and dexamethasone as induction therapy for newly diagnosed multiple myeloma patients destined for autologous stem-cell transplantation: MRC Myeloma IX randomized trial results. Haematologica 97, 442–450 (2012). 22058209 10.3324/haematol.2011.043372 PMC3291601

[b46] Merz M. . Subcutaneous versus intravenous bortezomib in two different induction therapies for newly diagnosed multiple myeloma: Interim analysis from the prospective GMMG-MM5 trial. Haematologica 100, 964–969 (2015). 25840597 10.3324/haematol.2015.124347 PMC4486231

[b47] Goldschmidt H. . Joint HOVON-50/GMMG-HD3 randomized trial on the effect of thalidomide as part of a high-dose therapy regimen and as maintenance treatment for newly diagnosed myeloma patients. Ann. Hematol. 82, 654–659 (2003). 12845480 10.1007/s00277-003-0685-2

[b48] Howie B. N., Donnelly P. & Marchini J. A flexible and accurate genotype imputation method for the next generation of genome-wide association studies. PLoS Genet. 5, e1000529 (2009). 19543373 10.1371/journal.pgen.1000529 PMC2689936

[b49] Delaneau O., Zagury J. F. & Marchini J. Improved whole-chromosome phasing for disease and population genetic studies. Nat. Methods 10, 5–6 (2013). 23269371 10.1038/nmeth.2307

[b50] R-Core-Team. R: A language and environment for statistical computing (R Foundation for Statistical Computing, Vienna, Austria 2013), http://www.R-project.org/.

[b51] Gogarten S. M. . GWASTools: an R/Bioconductor package for quality control and analysis of genome-wide association studies. Bioinformatics 28, 3329–3331 (2012). 23052040 10.1093/bioinformatics/bts610 PMC3519456

[b52] Weinhold N. . The 7p15.3 (rs4487645) association for multiple myeloma shows strong allele-specific regulation of the MYC-interacting gene CDCA7L in malignant plasma cells. Haematologica 100, e110–e113 (2015). 25480495 10.3324/haematol.2014.118786 PMC4349291

[b53] Consortium G. T. Human genomics. The Genotype-Tissue Expression (GTEx) pilot analysis: multitissue gene regulation in humans. Science 348, 648–660 (2015). 25954001 10.1126/science.1262110 PMC4547484

[b54] Westra H. J. . Systematic identification of trans eQTLs as putative drivers of known disease associations. Nat. Genet. 45, 1238–1243 (2013). 24013639 10.1038/ng.2756 PMC3991562

[b55] Nica A. C. . The architecture of gene regulatory variation across multiple human tissues: the MuTHER study. PLoS Genet. 7, e1002003 (2011). 21304890 10.1371/journal.pgen.1002003 PMC3033383

[b56] Zhang X. . Identification of common genetic variants controlling transcript isoform variation in human whole blood. Nat. Genet. 47, 345–352 (2015). 25685889 10.1038/ng.3220 PMC8273720

[b57] Gamazon E. R. . SCAN: SNP and copy number annotation. Bioinformatics 26, 259–262 (2010). 19933162 10.1093/bioinformatics/btp644 PMC2852202

[b58] Chiecchio L. . Deletion of chromosome 13 detected by conventional cytogenetics is a critical prognostic factor in myeloma. Leukemia 20, 1610–1617 (2006). 16826223 10.1038/sj.leu.2404304

[b59] Boyle E. M. . A molecular diagnostic approach able to detect the recurrent genetic prognostic factors typical of presenting myeloma. Genes Chromosomes Cancer 54, 91–98 (2015). 25287954 10.1002/gcc.22222 PMC4310140

[b60] Neben K. . Combining information regarding chromosomal aberrations t(4;14) and del(17p13) with the International Staging System classification allows stratification of myeloma patients undergoing autologous stem cell transplantation. Haematologica 95, 1150–1157 (2010). 20220069 10.3324/haematol.2009.016436 PMC2895040

[b61] Boyle A. P. . Annotation of functional variation in personal genomes using RegulomeDB. Genome Res. 22, 1790–1797 (2012). 22955989 10.1101/gr.137323.112 PMC3431494

[b62] Ward L. D. & Kellis M. HaploReg: a resource for exploring chromatin states, conservation, and regulatory motif alterations within sets of genetically linked variants. Nucleic Acids Res. 40, D930–D934 (2012). 22064851 10.1093/nar/gkr917 PMC3245002

[b63] Cooper G. M. . Single-nucleotide evolutionary constraint scores highlight disease-causing mutations. Nat. Methods 7, 250–251 (2010). 20354513 10.1038/nmeth0410-250 PMC3145250

