# Supplementary material for: Genome-wide association study identifies variation at 6q25.1 associated with survival in multiple myeloma
Source: Nat Commun. 2016 Jan 8;7:10290. doi: 10.1038/ncomms10290 (PMC4729868; doi:10.1038/ncomms10290)
Supplement: Supplementary Information — Supplementary Figures 1-21, Supplementary Tables 1-6 and Supplementary References. [file ncomms10290-s1.pdf]

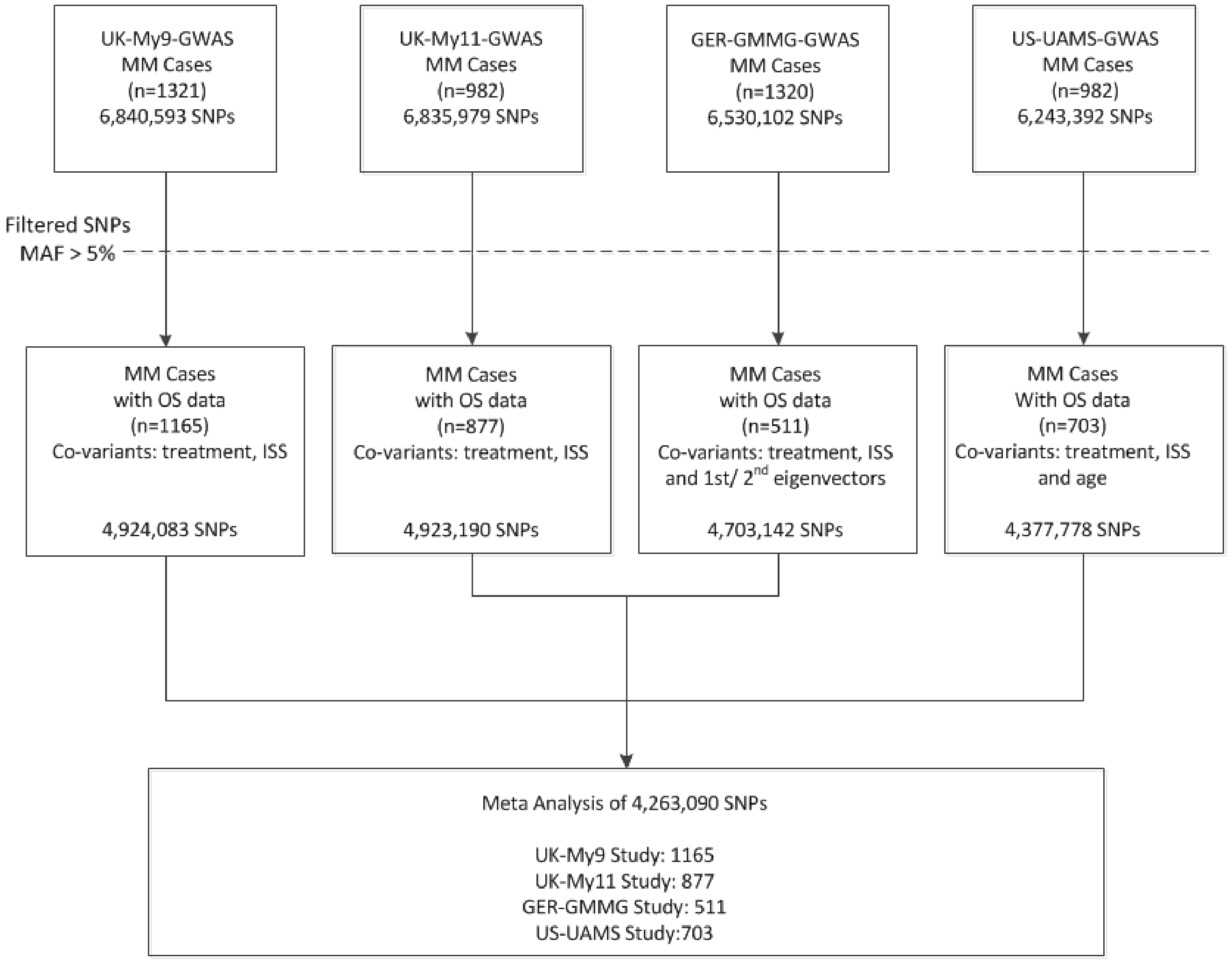

Supplementary Figure 1: GWAS data quality control.

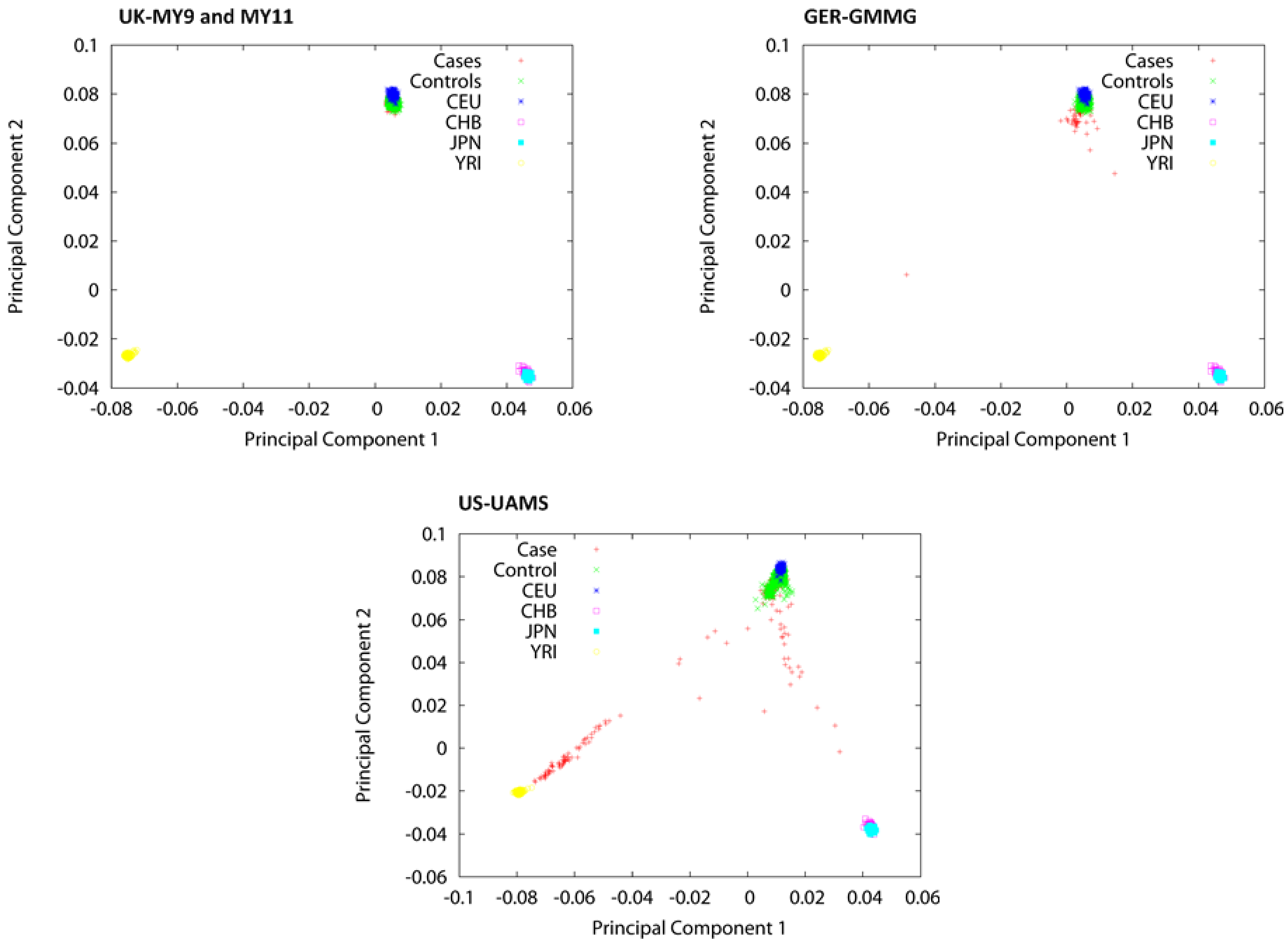

Supplementary Figure 2: Identification of samples of non-European origin. The first two principal components of the analysis were plotted for German-GWA. (a) HapMap CEU individuals are plotted in blue; CHB+JPT individuals are plotted in green; YRI individuals are plotted in red; GWAS cases are plotted in black and controls are plotted in purple. (b) The same plot is shown after the removal of cases and controls of non-European origin.

UK-My9

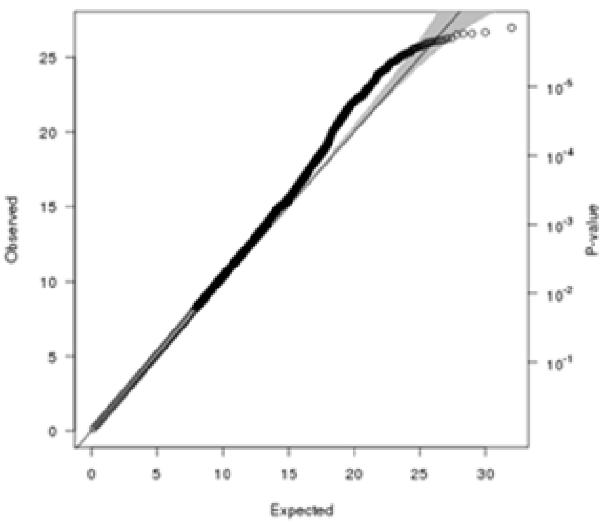

UK-My11

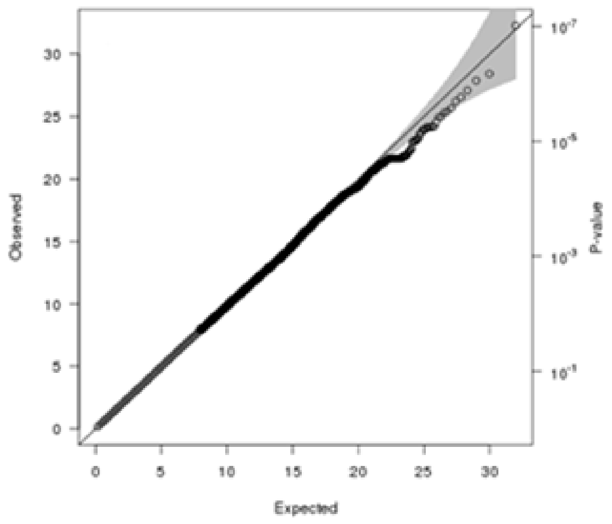

GER-GMMG

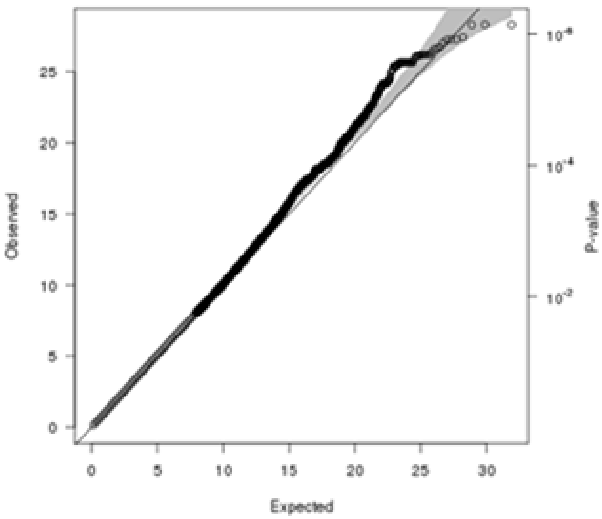

λ =1.02, n=4,703,142

US-UAMS

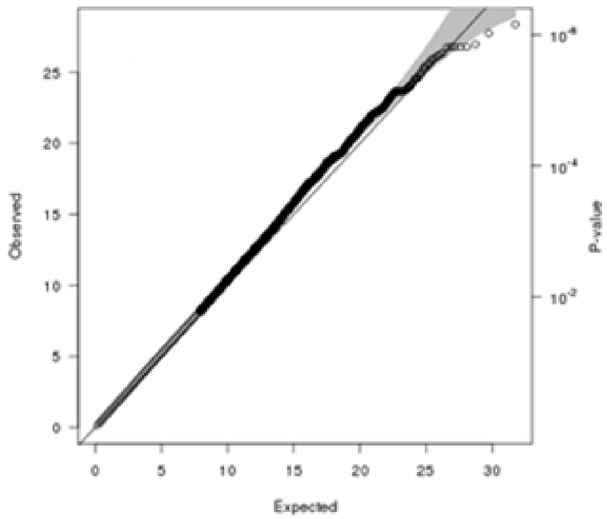

λ =1.03, n=4,377,778

Supplementary Figure 3: Quantile-Quantile (Q-Q) plot for the four patient cohort analyses. The y-axis corresponds to the observed -$\log_{10}$ *P*-value, and the x-axis the expected -$\log_{10}$ *P*-value. The red line represents the expected distribution under the null hypothesis of no association. All statistical tests were two-sided.

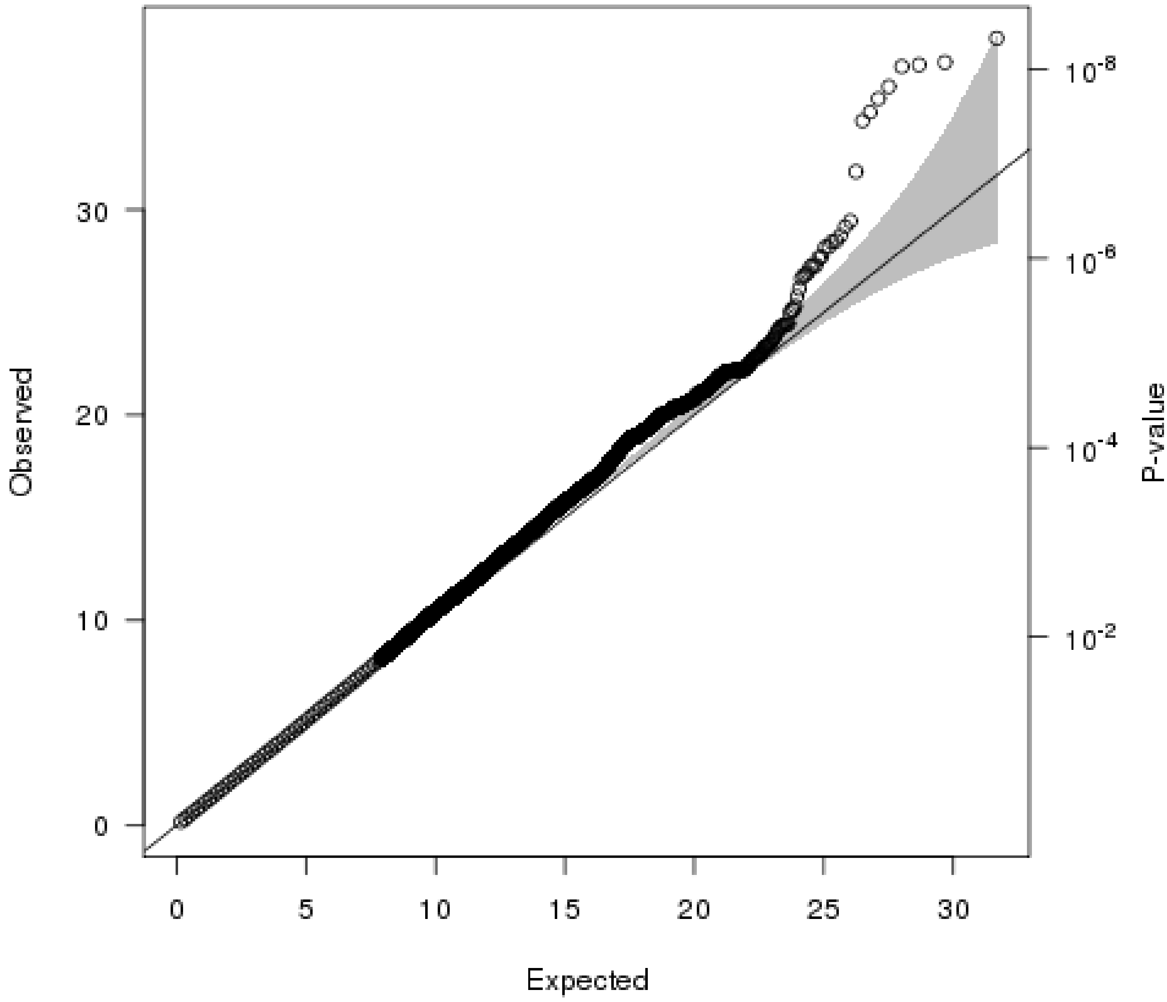

λ =1.01, n=4,263,089

Supplementary Figure 4: Quantile-Quantile (Q-Q) plot for the for the combined analyses. The y-axis corresponds to the observed -$\log_{10}$ *P*-value, and the x-axis the expected -$\log_{10}$ *P*-value. The red line represents the expected distribution under the null hypothesis of no association. All statistical tests were two-sided.

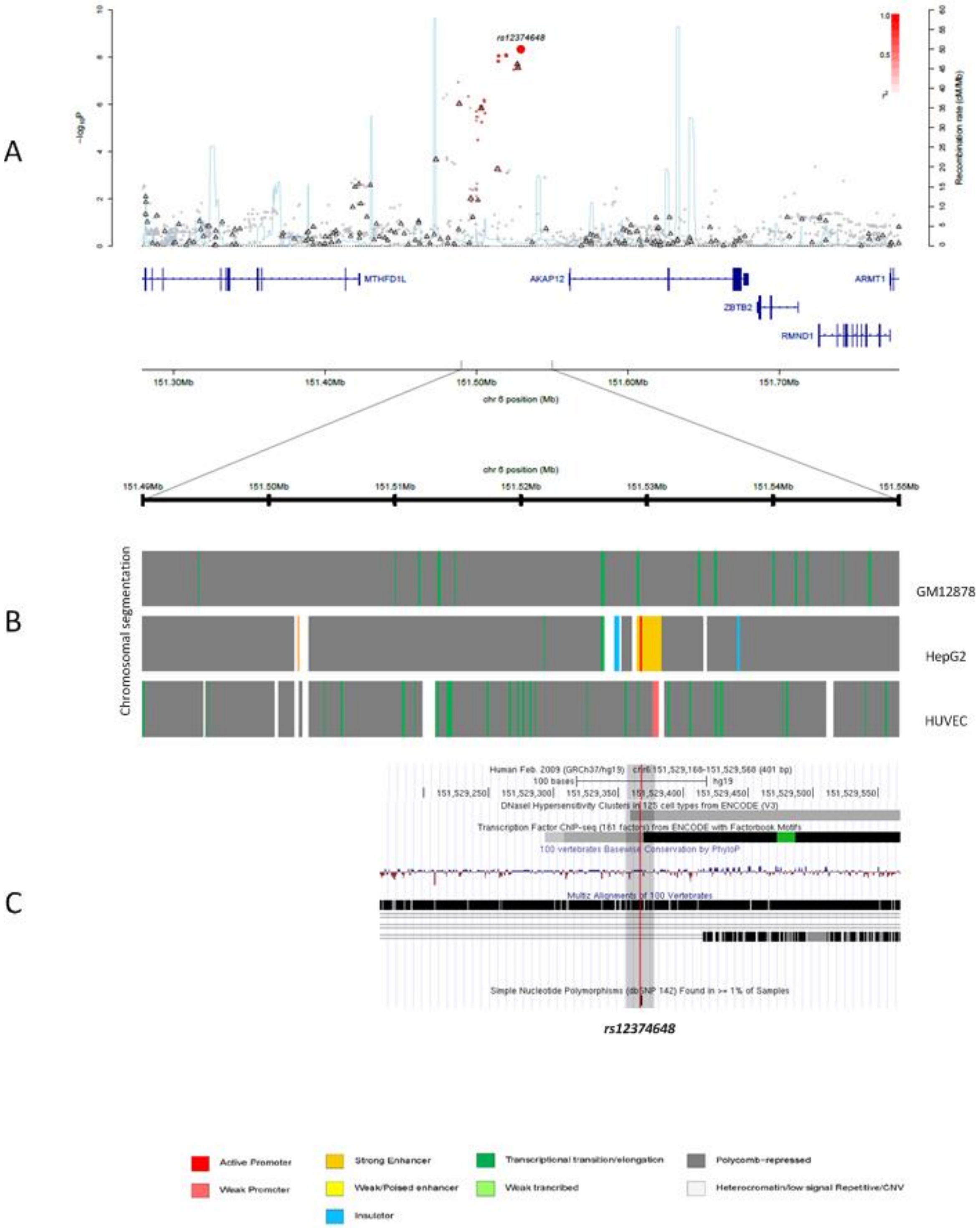

Supplementary Figure 5: Regional plot of association results and recombination rates for the rs12374648 (6q25.1) MM-OS risk locus annotated with ENCODE data. A) Plots show association results of both genotyped (triangles) and imputed (circles) SNPs in the GWAS samples and recombination rates. $-\log_{10}$ *P* values (*y* axes) of the SNPs are shown according to their chromosomal positions (*x* axes). B) Chromatin state segmentation tracks (Combined) for Lymphoblast (GM12878), human liver carcinoma (HepG2) and Human Umbilical Vein Endothelial Cells (HUVEC) cell lines, data derived from the ENCODE project. C. RegulomeDB results for rs12374648**.**

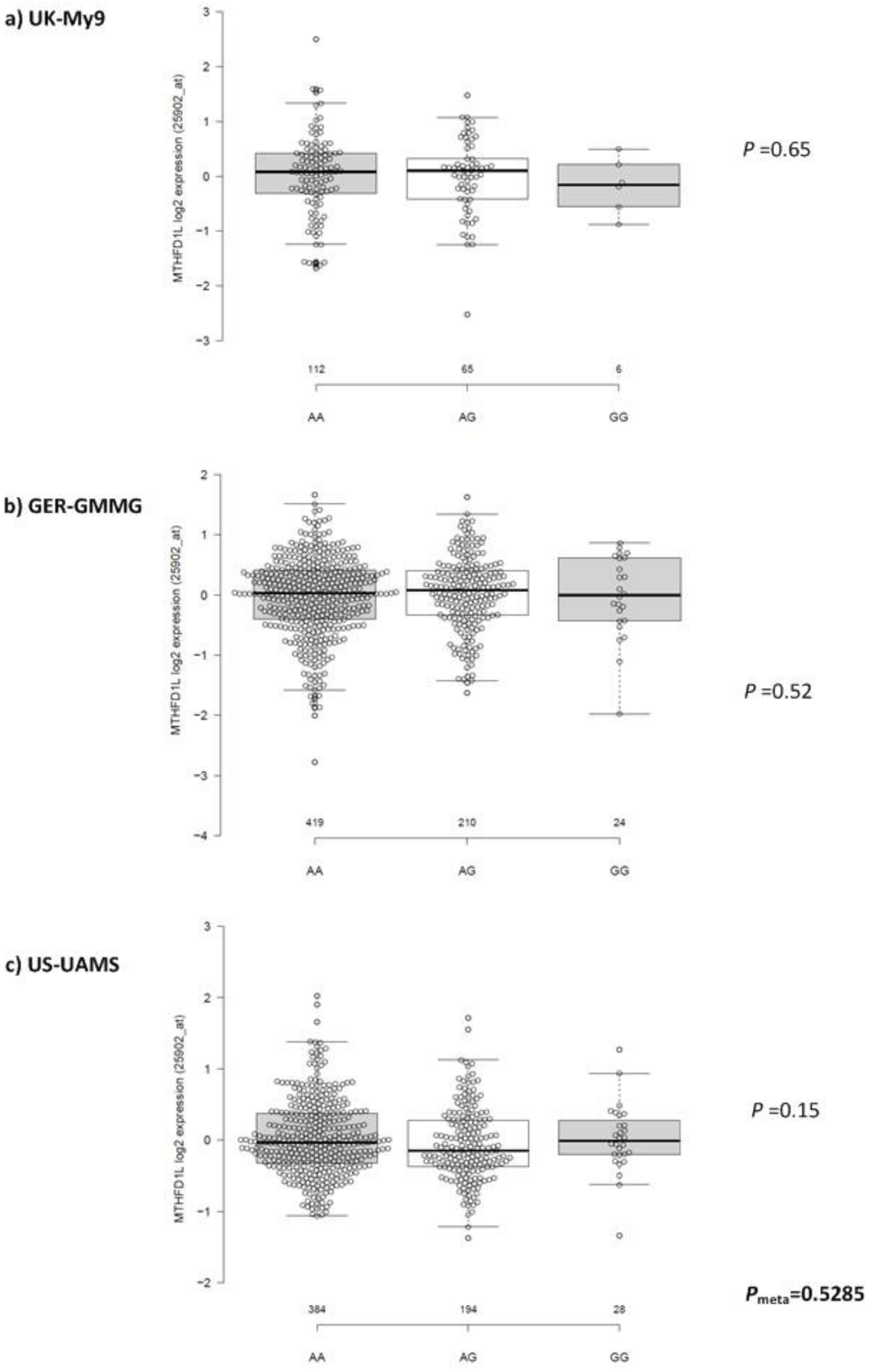

Supplementary Figure 6: Relationship between rs12374648 genotypes and expression at *MTHFD1L*. Box plots show relationship of SNP genotype with Log_2 (gene expression). The central line in each box is the median, the bottom and top of the box are the first and third quartiles. The notches on each box are +/-1.58* interquartile range/sqrt(n), defining the 95% confidence where the median are different. Bee swarm arranged circles are the data-points.

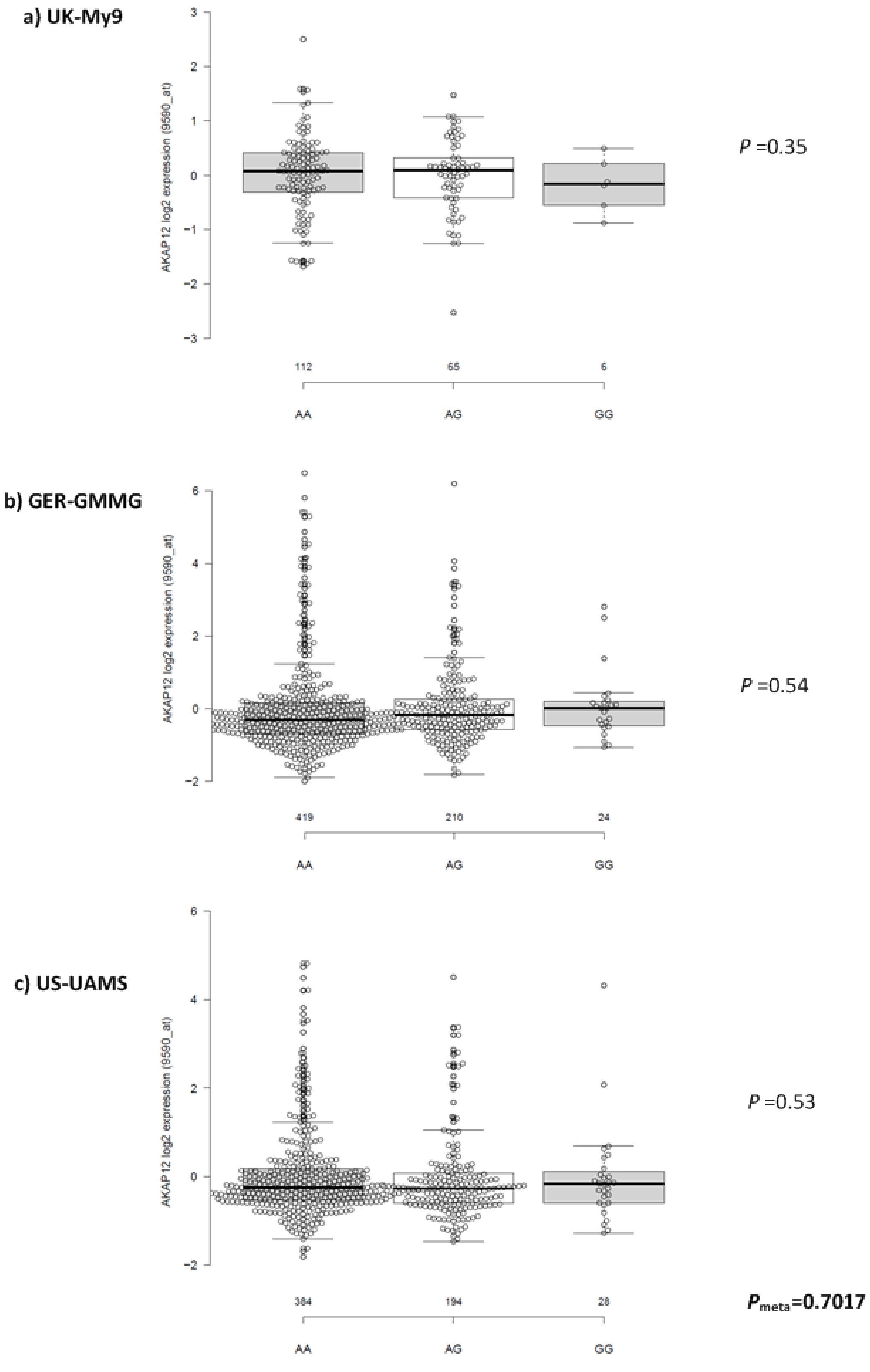

Supplementary Figure 7: Relationship between rs12374648 and expression at *AKAP12*. Box plots show relationship of SNP genotype with Log_2 (gene expression). The central line in each box is the median, the bottom and top of the box are the first and third quartiles. The notches on each box are +/-1.58* interquartile range/sqrt(n), defining the 95% confidence where the median are different. Bee swarm arranged circles are the data-points.

a) *AKAP12* (cg25529303)

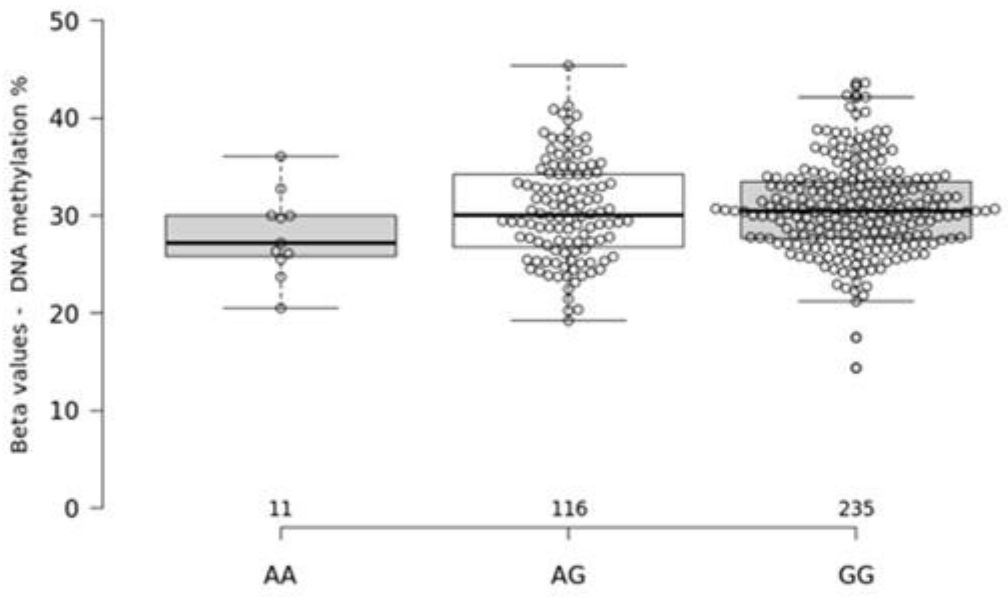

***P*=0.0097**

b) *MTHFD1L* (cg09070799)

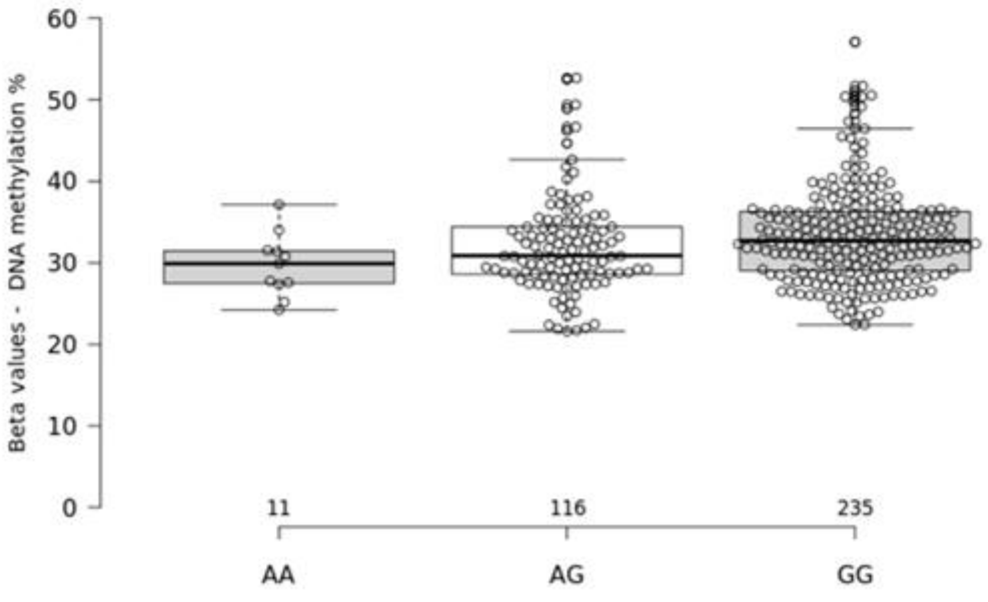

***P*=0.0077**

Supplementary Figure 8: Relationship between rs12374648 and DNA methylation at 6p25.1. Box plots show relationship of SNP genotype with Log_2 (DNA methylation) in the UK-My11 patients. The central line in each box is the median, the bottom and top of the box are the first and third quartiles. The notches on each box are +/-1.58* interquartile range/sqrt(n), defining the 95% confidence where the median are different. Bee swarm arranged circles are the data-points.

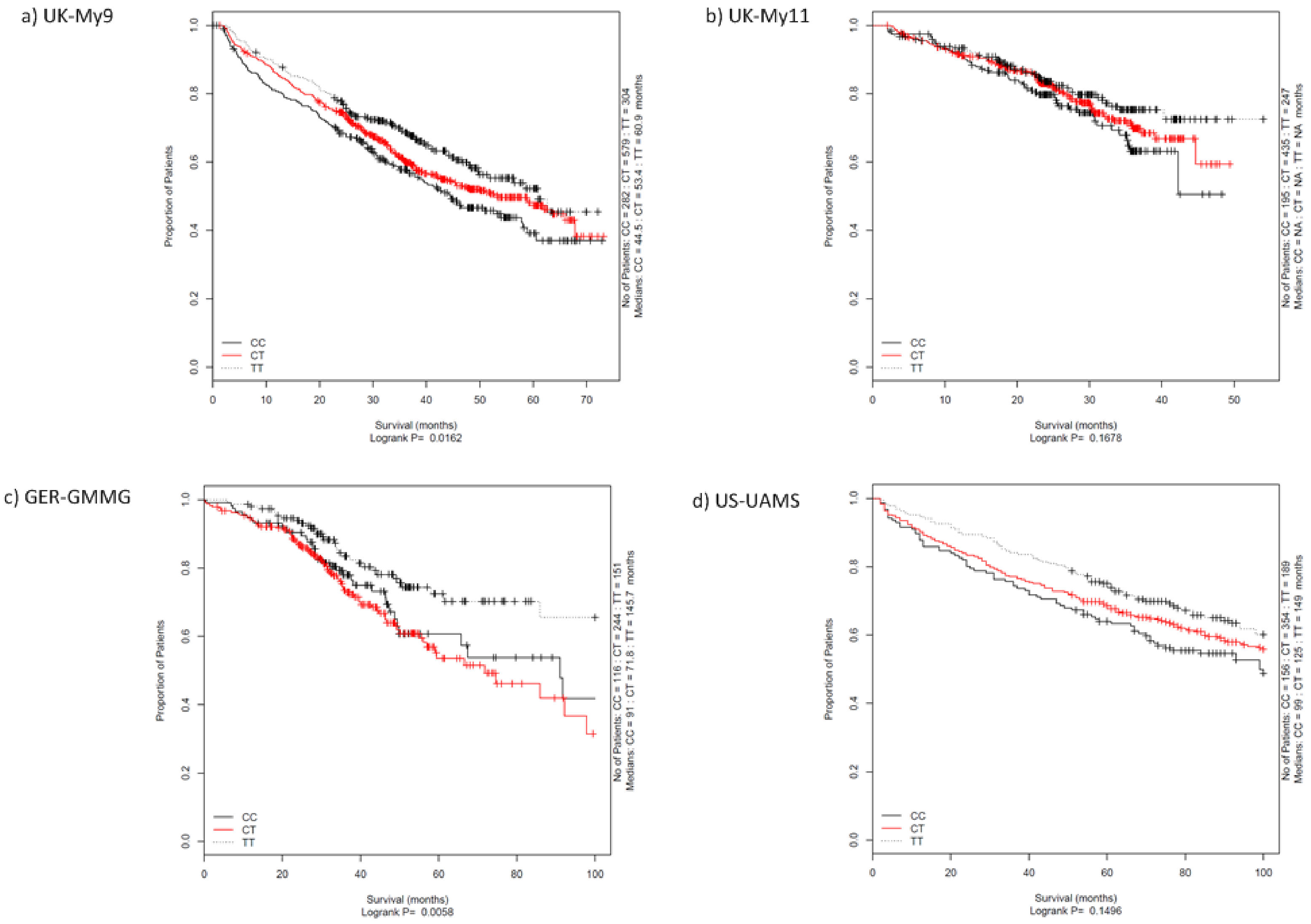

Supplementary Figure 9: Kaplan–Meier curves for 1q23.3 (rs1934908) and MM-OS. Vertical ticks indicate censored data-points.

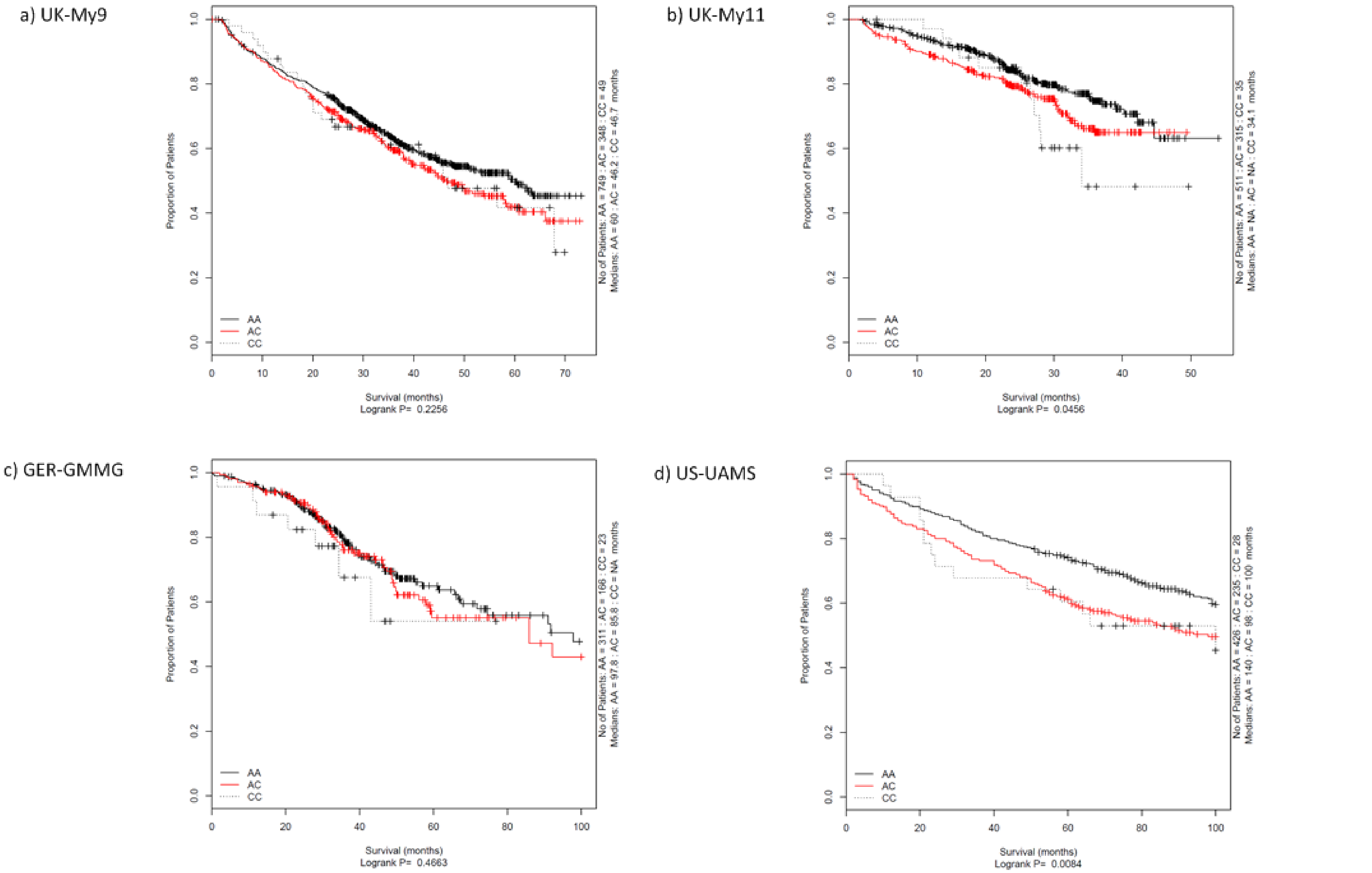

Supplementary Figure 10: Kaplan–Meier curves for 19q13.11 (rs1974807) for MM-OS. Vertical ticks indicate censored data-points.

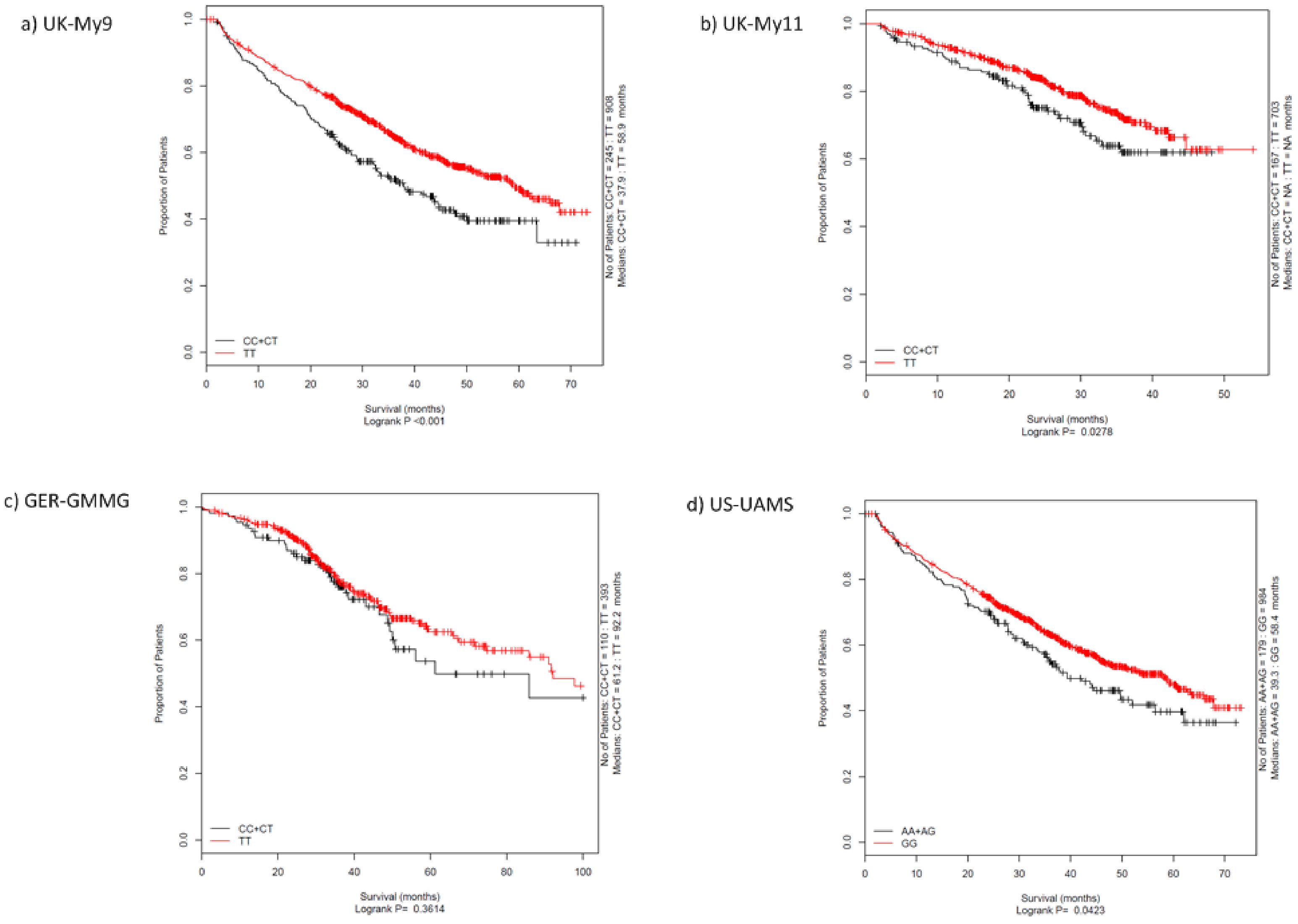

Supplementary Figure 11: Kaplan–Meier curves for 3q13.31 (rs4682170) and MM-OS. Vertical ticks indicate censored data-points.

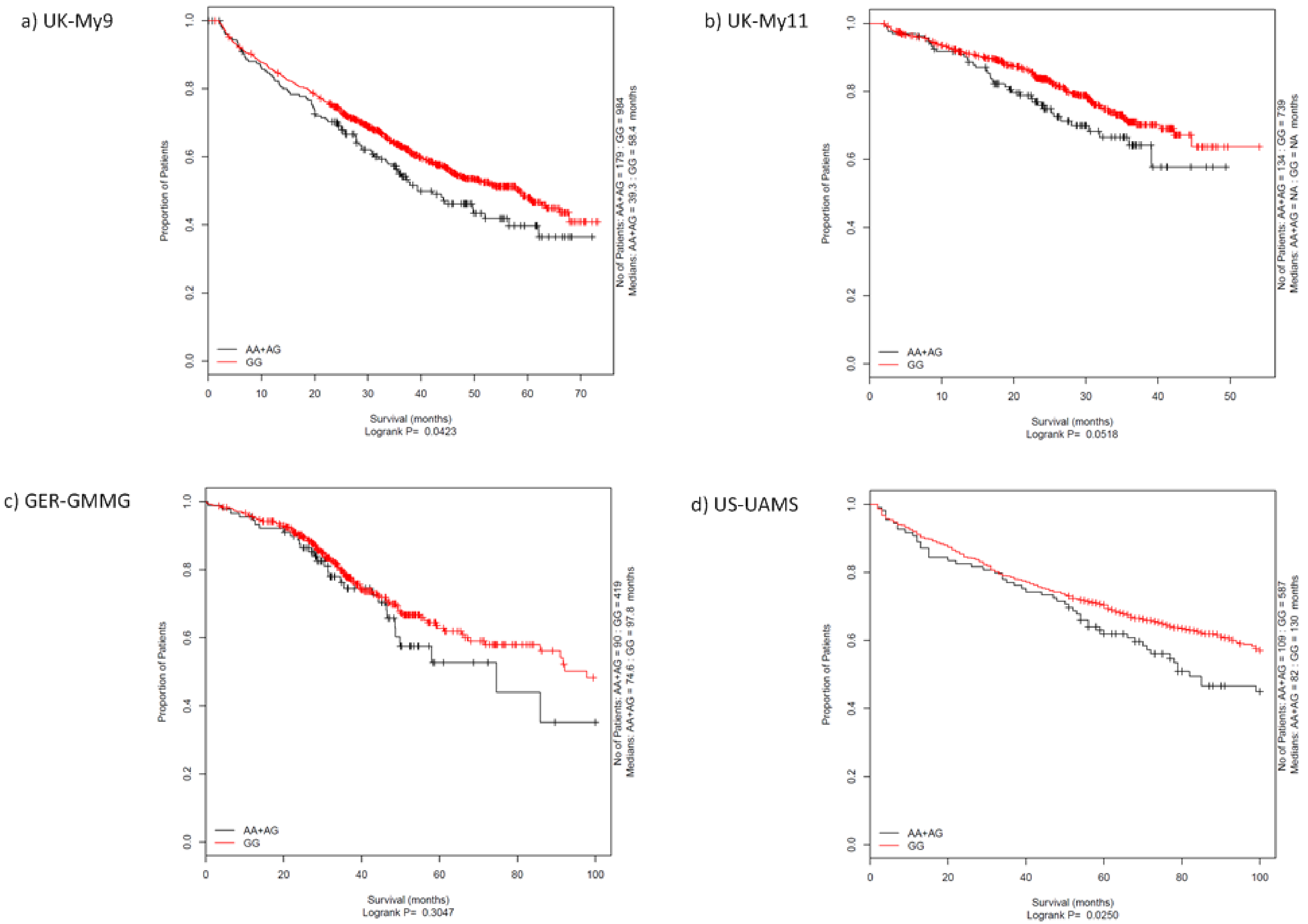

Supplementary Figure 12: Kaplan–Meier curves for 18q21.32 (rs57942319) for MM-OS. Vertical ticks indicate censored data-points.

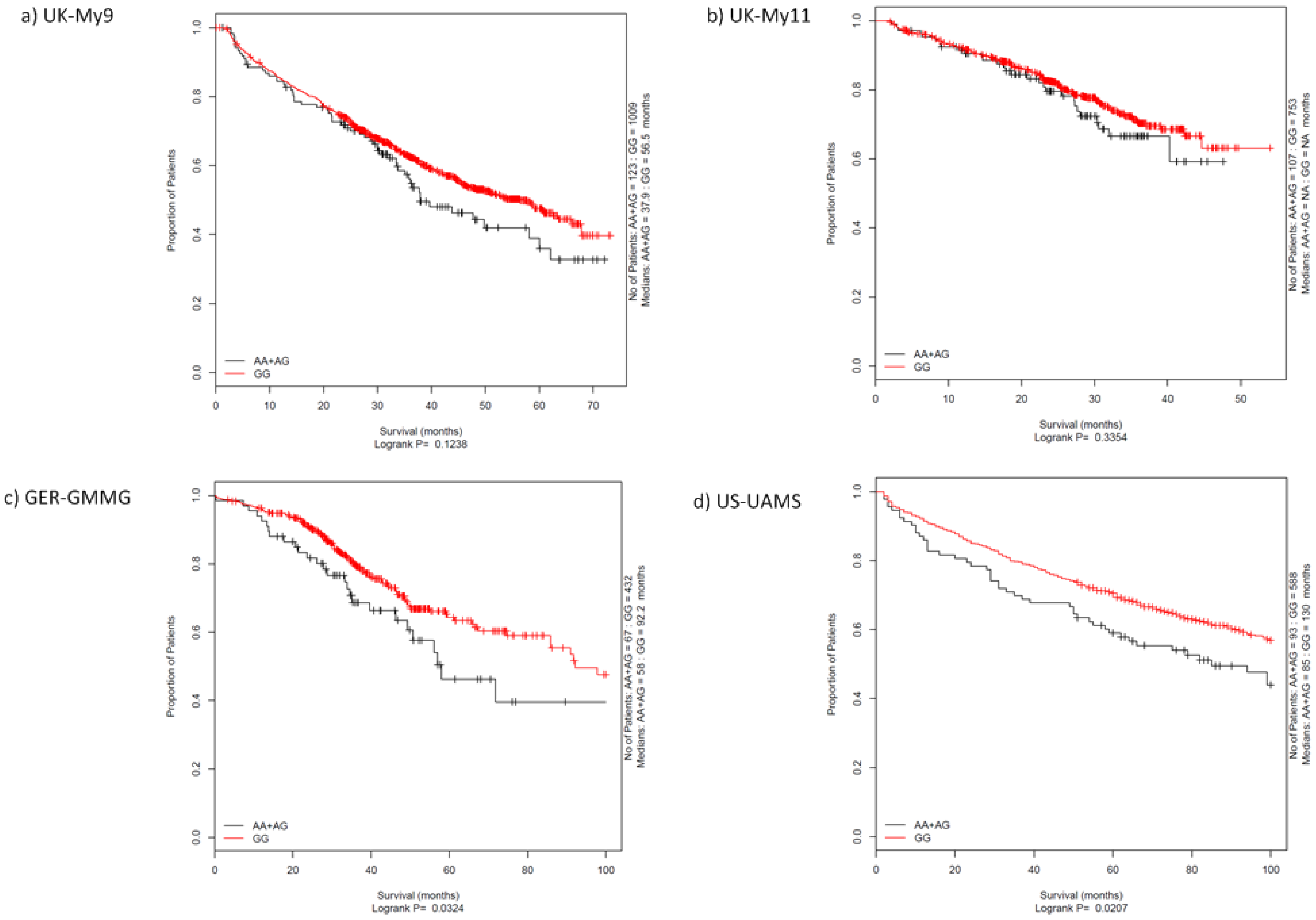

Supplementary Figure 13: Kaplan–Meier curves for 2q22.1 (rs61070260) and MM-OS. Vertical ticks indicate censored data-points.

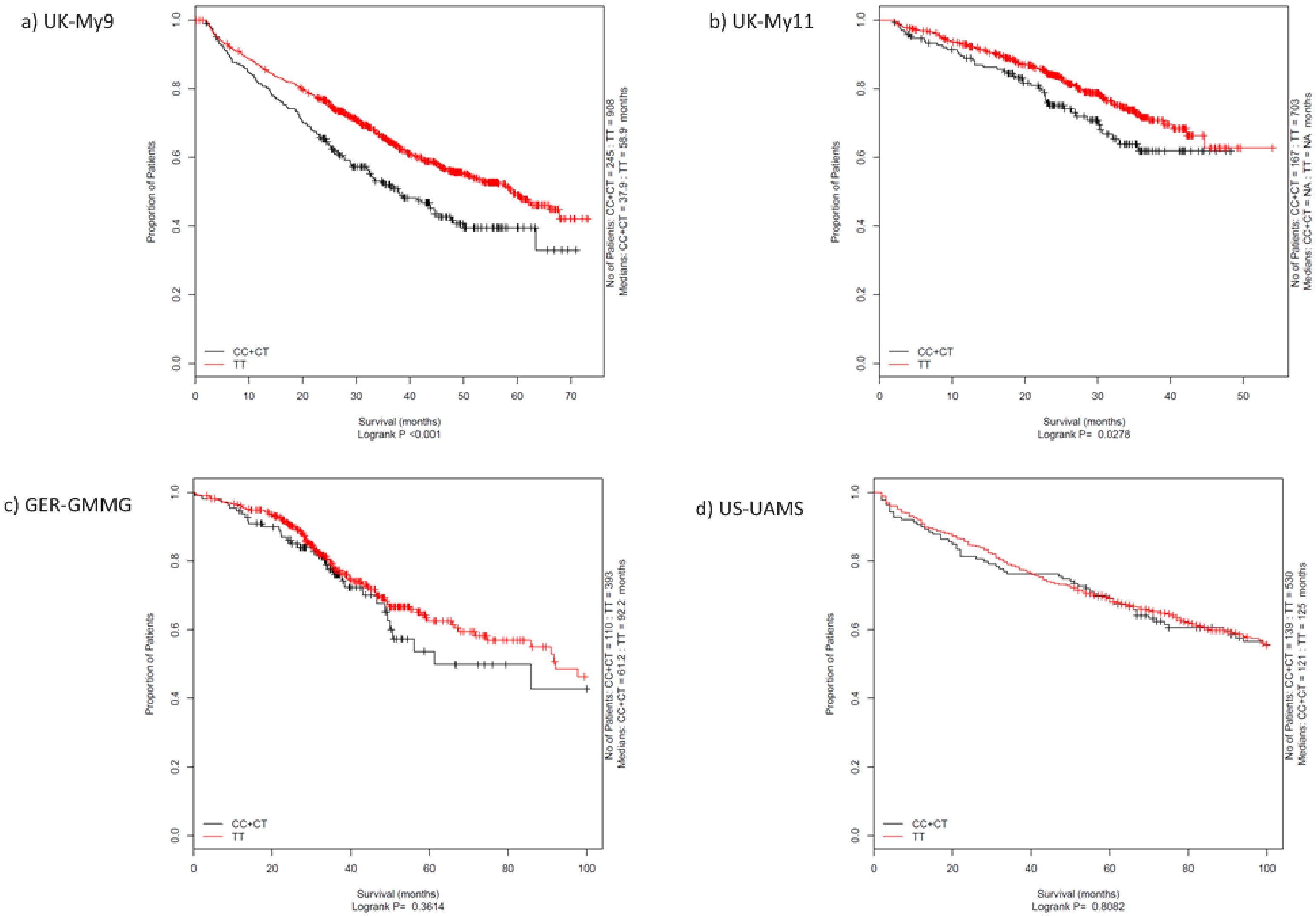

Supplementary Figure 14: Kaplan–Meier curves for 5q31.3 (rs2906053) and MM-OS. Vertical ticks indicate censored data-points.

1q23.3 (rs1934908)

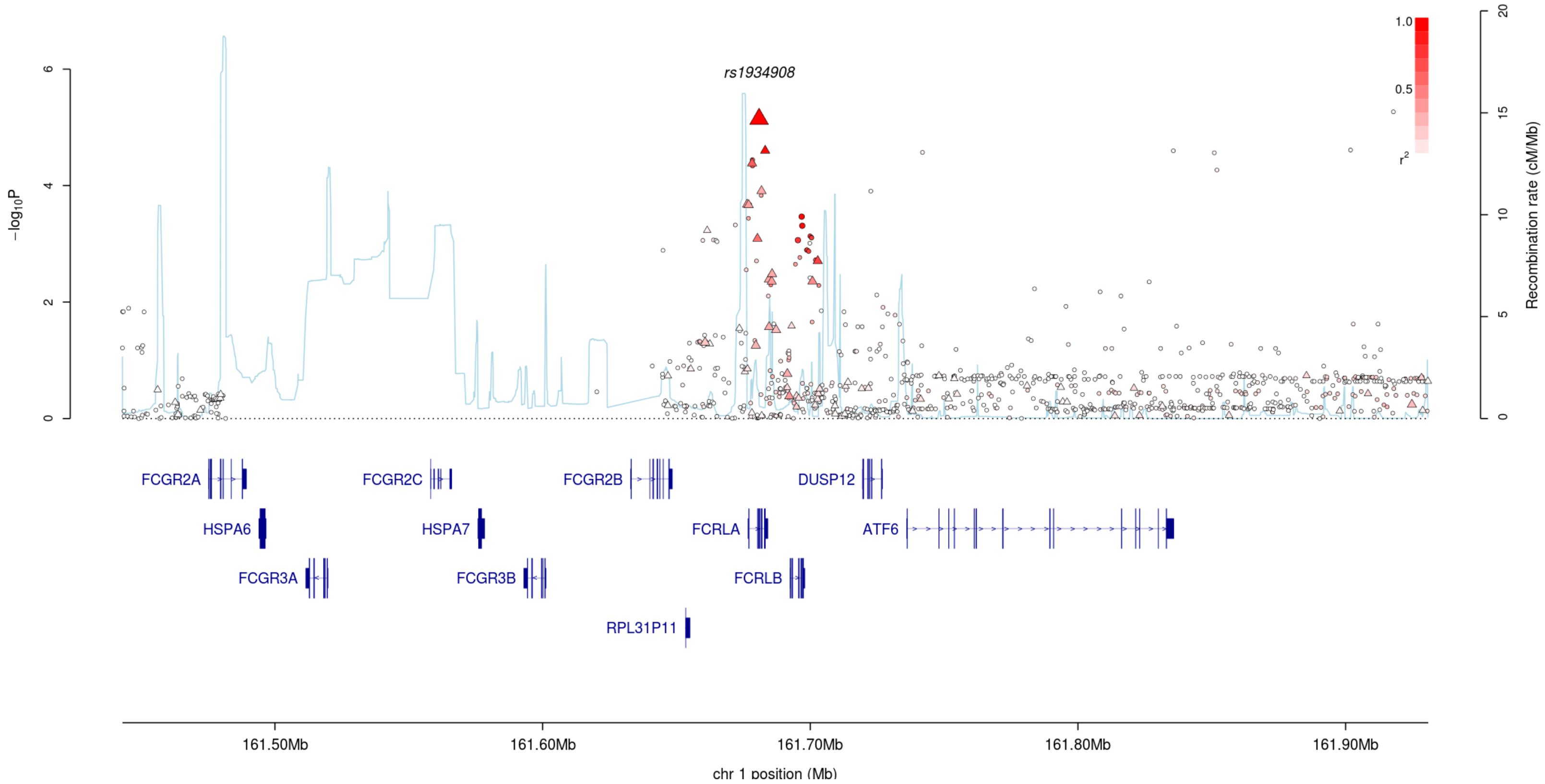

Supplementary Figure 15: Regional plots of association results and recombination rates for the 1q23.3 (rs1934908) and 19q13.11 (rs197480) risk loci. Plots show association results of both genotyped (triangles) and imputed (circles) SNPs in the GWAS samples and recombination rates. $-\log_{10}$ *P* values (*y* axes) of the SNPs are shown according to their chromosomal positions (*x* axes). The top genotyped SNP in each combined analysis is shown as a large diamond and is labeled by its rsID. The color intensity of each symbol reflects the extent of LD with the top genotyped SNP, white (r^2 = 0) through to dark red (r^2 = 1.0). Genetic recombination rates, estimated using HapMap samples from Utah residents of western and northern European ancestry (CEU), are shown with a light blue line. Physical positions are based on NCBI build 37 of the human genome. Also shown are the relative positions of genes and transcripts mapping to the region of association. Genes have been redrawn to show their relative positions; therefore, maps are not to physical scale.

2q22.1 (rs61070260)

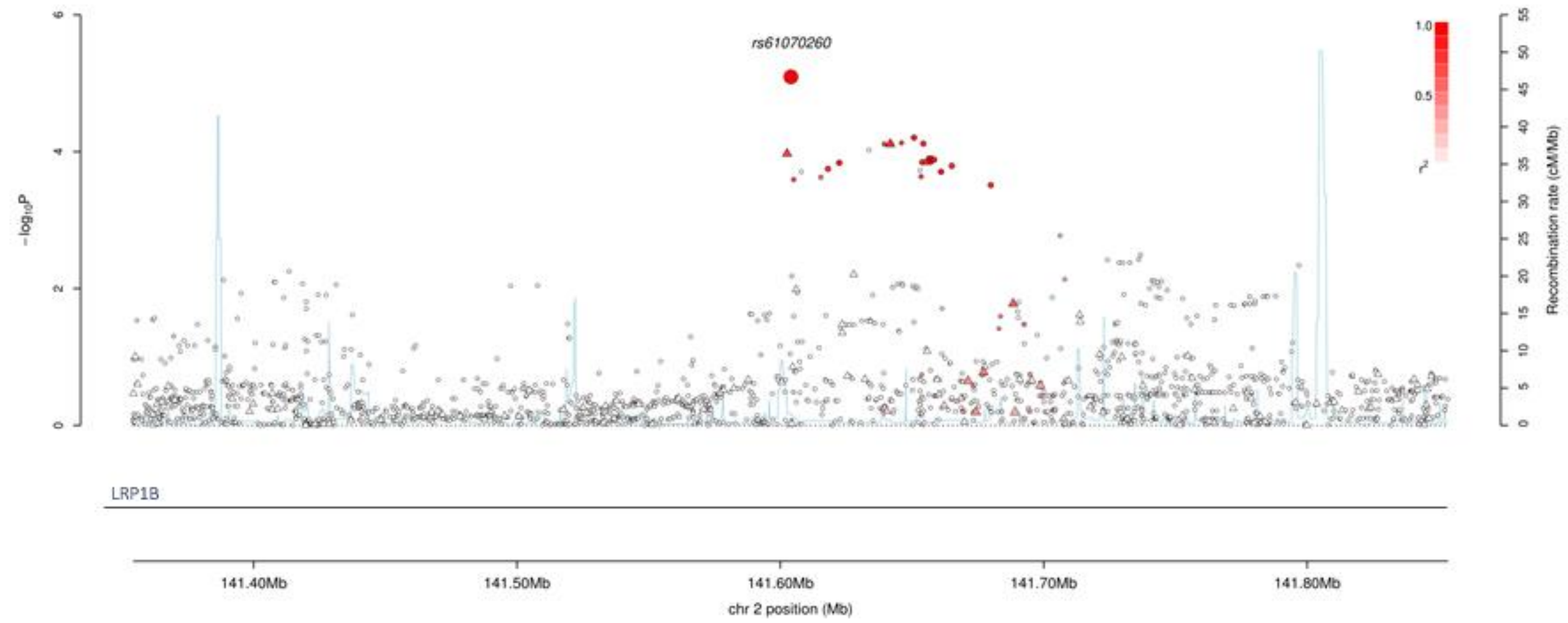

Supplementary Figure 16: Regional plots of association results and recombination rates for 2q22.1 (rs61070260) risk loci. Plots show association results of both genotyped (triangles) and imputed (circles) SNPs in the GWAS samples and recombination rates. $-\log_{10}$ *P* values (*y* axes) of the SNPs are shown according to their chromosomal positions (*x* axes). The top genotyped SNP in each combined analysis is shown as a large diamond and is labeled by its rsID. The color intensity of each symbol reflects the extent of LD with the top genotyped SNP, white (r^2 = 0) through to dark red (r^2 = 1.0). Genetic recombination rates, estimated using HapMap samples from Utah residents of western and northern European ancestry (CEU), are shown with a light blue line. Physical positions are based on NCBI build 37 of the human genome. Also shown are the relative positions of genes and transcripts mapping to the region of association. Genes have been redrawn to show their relative positions; therefore, maps are not to physical scale.

3q13.31 (rs4682170)

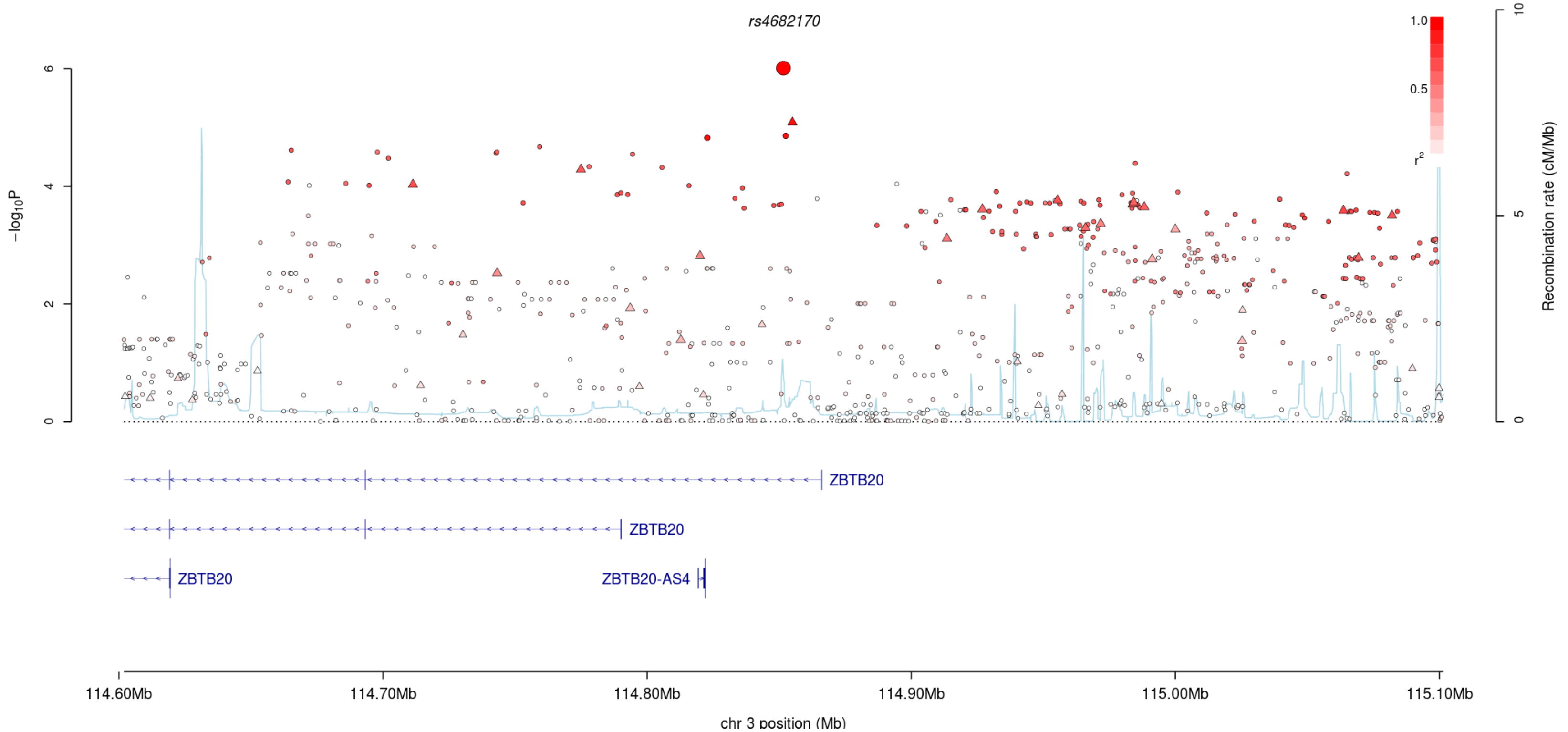

Supplementary Figure 17: Regional plots of association results and recombination rates for the 3q13.31 (rs4682170) and 5q31.3 (rs2906053) risk loci. Plots show association results of both genotyped (triangles) and imputed (circles) SNPs in the GWAS samples and recombination rates. $-\log_{10}$ *P* values (*y* axes) of the SNPs are shown according to their chromosomal positions (*x* axes). The top genotyped SNP in each combined analysis is shown as a large diamond and is labeled by its rsID. The color intensity of each symbol reflects the extent of LD with the top genotyped SNP, white (r^2 = 0) through to dark red (r^2 = 1.0). Genetic recombination rates, estimated using HapMap samples from Utah residents of western and northern European ancestry (CEU), are shown with a light blue line. Physical positions are based on NCBI build 37 of the human genome. Also shown are the relative positions of genes and transcripts mapping to the region of association. Genes have been redrawn to show their relative positions; therefore, maps are not to physical scale.

5q31.3 (rs2906053)

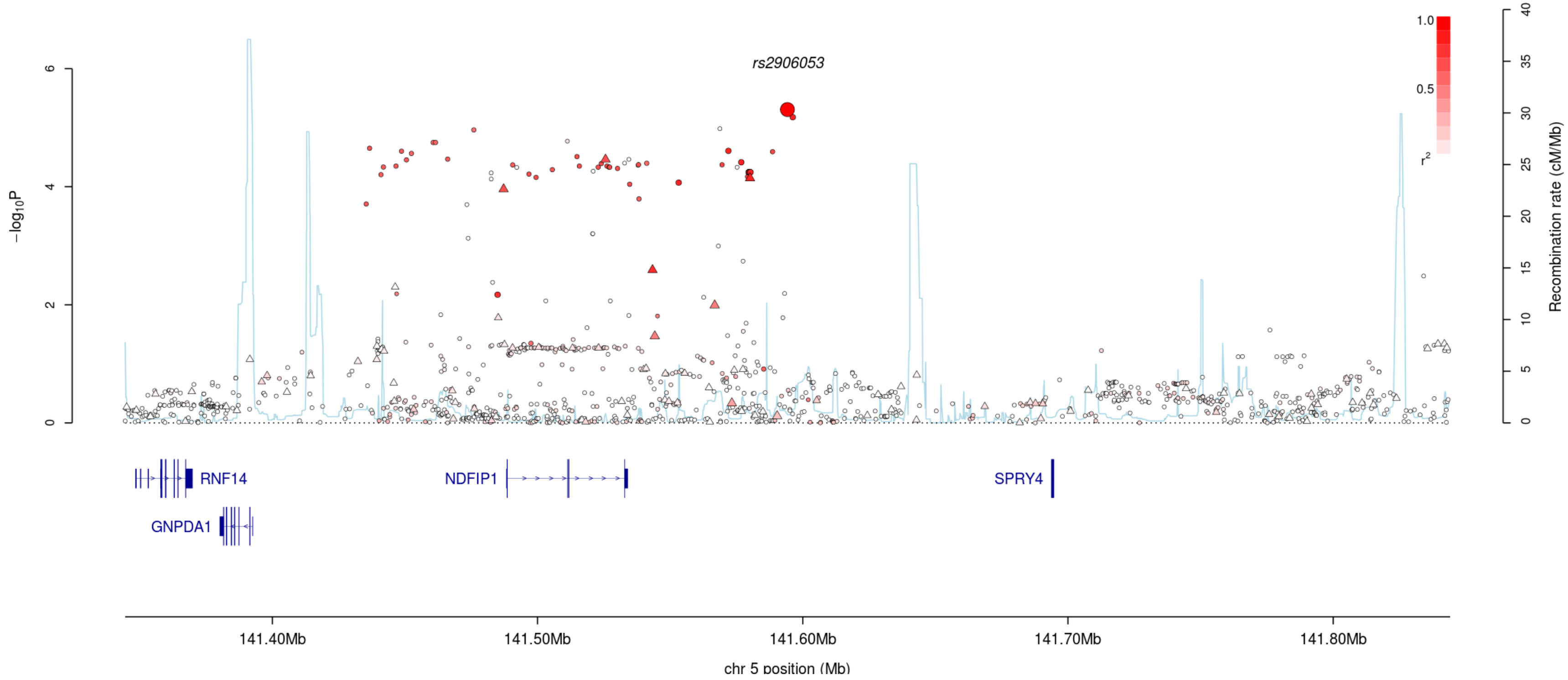

Supplementary Figure 18: Regional plots of association results and recombination rates for 5q31.3 (rs2906053) risk loci. Plots show association results of both genotyped (triangles) and imputed (circles) SNPs in the GWAS samples and recombination rates. $-\log_{10}$ *P* values (*y* axes) of the SNPs are shown according to their chromosomal positions (*x* axes). The top genotyped SNP in each combined analysis is shown as a large diamond and is labeled by its rsID. The color intensity of each symbol reflects the extent of LD with the top genotyped SNP, white (r^2 = 0) through to dark red (r^2 = 1.0). Genetic recombination rates, estimated using HapMap samples from Utah residents of western and northern European ancestry (CEU), are shown with a light blue line. Physical positions are based on NCBI build 37 of the human genome. Also shown are the relative positions of genes and transcripts mapping to the region of association. Genes have been redrawn to show their relative positions; therefore, maps are not to physical scale.

18q21.32 (rs57942319)

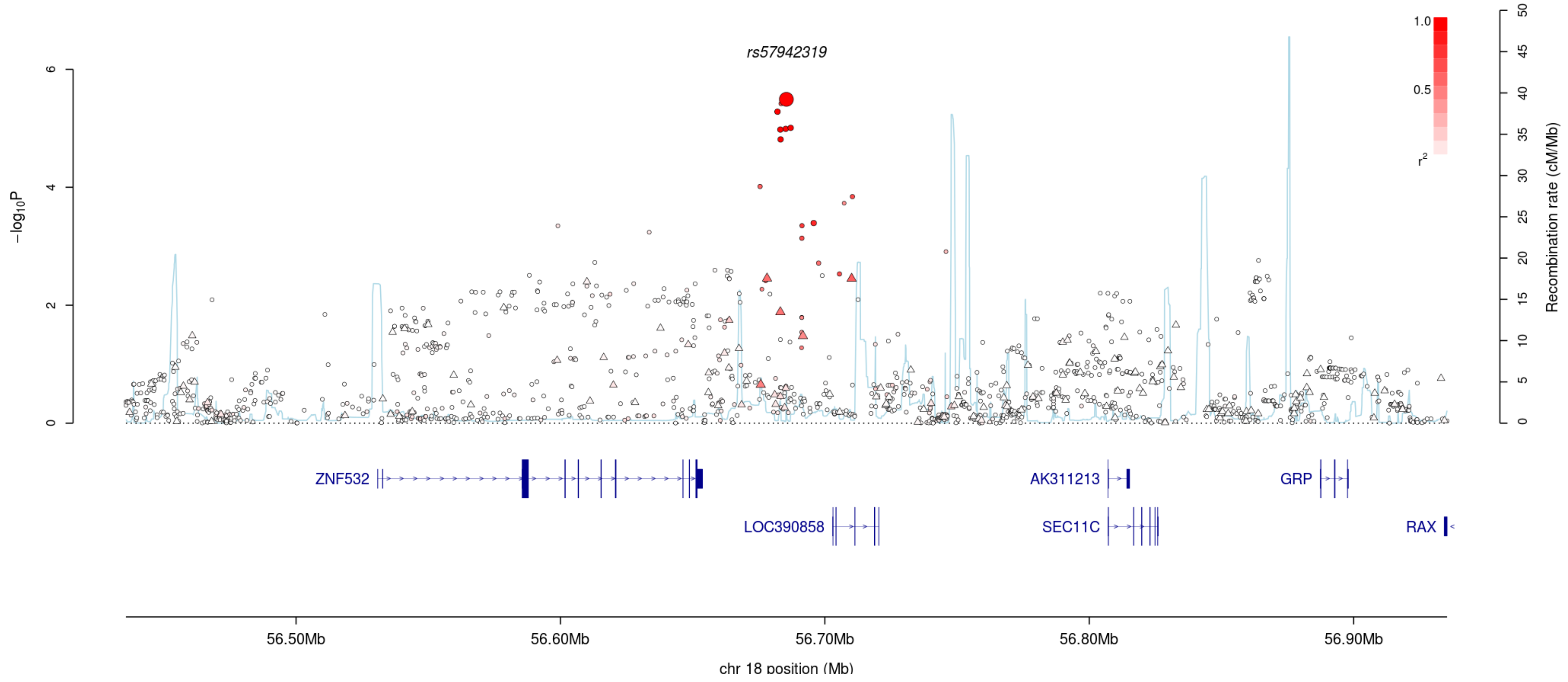

Supplementary Figure 19: Regional plots of association results and recombination rates for the 18q21.32 (rs57942319) and 2q22.1 (rs61070260) risk loci. Plots show association results of both genotyped (triangles) and imputed (circles) SNPs in the GWAS samples and recombination rates. $-\log_{10}$ *P* values (*y* axes) of the SNPs are shown according to their chromosomal positions (*x* axes). The top genotyped SNP in each combined analysis is shown as a large diamond and is labeled by its rsID. The color intensity of each symbol reflects the extent of LD with the top genotyped SNP, white (r^2 = 0) through to dark red (r^2 = 1.0). Genetic recombination rates, estimated using HapMap samples from Utah residents of western and northern European ancestry (CEU), are shown with a light blue line. Physical positions are based on NCBI build 37 of the human genome. Also shown are the relative positions of genes and transcripts mapping to the region of association. Genes have been redrawn to show their relative positions; therefore, maps are not to physical scale.

19q13.11 (rs1974807)

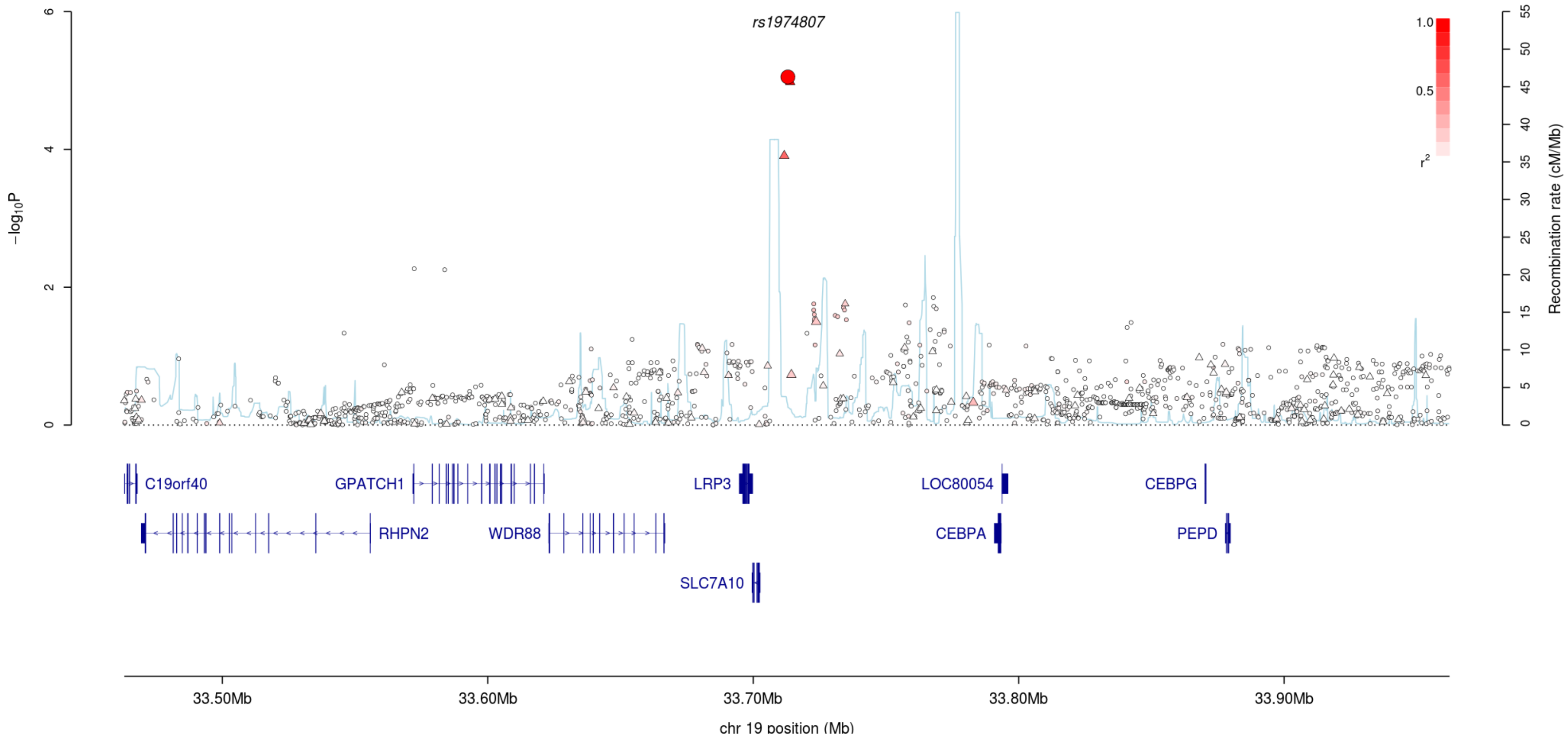

Supplementary Figure 20: Regional plots of association results and recombination rates for the 19q13.11 (rs197480) risk loci. Plots show association results of both genotyped (triangles) and imputed (circles) SNPs in the GWAS samples and recombination rates. $-\log_{10}$ *P* values (*y* axes) of the SNPs are shown according to their chromosomal positions (*x* axes). The top genotyped SNP in each combined analysis is shown as a large diamond and is labeled by its rsID. The color intensity of each symbol reflects the extent of LD with the top genotyped SNP, white (r^2 = 0) through to dark red (r^2 = 1.0). Genetic recombination rates, estimated using HapMap samples from Utah residents of western and northern European ancestry (CEU), are shown with a light blue line. Physical positions are based on NCBI build 37 of the human genome. Also shown are the relative positions of genes and transcripts mapping to the region of association. Genes have been redrawn to show their relative positions; therefore, maps are not to physical scale.

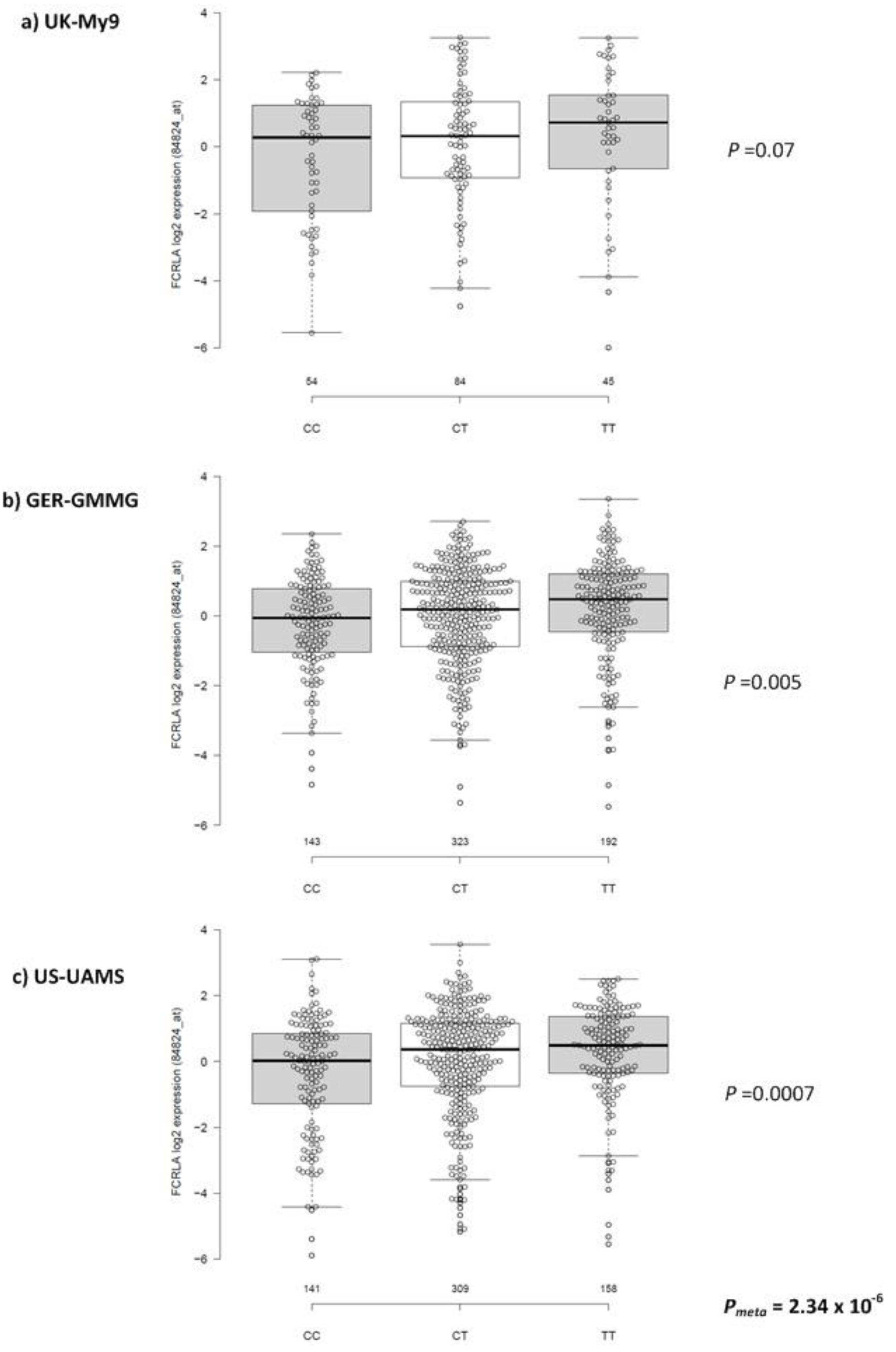

Supplementary Figure 21: Relationship between Myeloma OS SNP rs1934908 and expression *FCRLA*. Box plots show relationship of SNP genotype with Log_2 (gene expression). The central line in each box is the median, the bottom and top of the box are the first and third quartiles. The notches on each box are +/-1.58* interquartile range/sqrt(n), defining the 95% confidence where the median are different. Bee swarm arranged circles are the data-points.

Univariate - UK-My9

Chromsomal aberration	NA[a]	HR[b] (95% CI)[c]	*P*
Hyperdiploidy	529	0.76 (0.6-0.96)	0.02
t4;14	500	1.59 (1.14-2.2)	0.006
t6;14	513	0.89 (0.28-2.77)	0.834
t11;14	501	0.92 (0.66-1.28)	0.6
t14;16	503	2.11 (1.27-3.5)	0.004
t14;20	508	2.73 (1.45-5.14)	0.002
Gain 1q21	599	1.74 (1.35-2.23)	1.95×10^{-5}
Del p53	522	1.87 (1.33-2.63)	0.0004
rs12374648 - G	0	1.33 (1.14-1.55)	0.0003
rs12374648 - G -adjusted for ISS and treatment	0	1.45 (1.24-1.68)	1.69×10^{-6}

Multivariate - UK-My9

Chromsomal aberration	HR (95% CI)	*P*
t4;14	1.48 (1.01-2.18)	0.044
t14;16	2.83 (1.59-5.04)	4.1×10^{-4}
t14;20	2.30 (1.06-4.99)	0.03
Gain 1q21	1.48 (1.11-1.97)	0.007
Del p53	1.88 (1.27-2.78)	0.002
rs12374648 - G	1.48 (1.18-1.85)	6.8×10^{-4}

[a]Cases with missing data, [b]Hazards ratio. [c]95% Confidence Interval.

Supplementary Table 1: Univariate and Multivariate Cox proportional hazards model of MM-OS at 6q25.1 (rs12374648) with major chromosomal aberrations in the UK-My9 cohort.

SNP	Base pair	Risk allele	Study	RAF[a]	N	Events	Genoytypes			HR[b] (95%CI)[c]	*P*
**rs1934908(1q23.3)**	**161680868**	**C**					**CC**	**CT**	**TT**		
			UK-My9	0.49	1165	512	282	579	304	1.19 (1.05-1.34)	0.0059
			UK-My11	0.47	877	201	195	435	247	1.17 (0.96-1.42)	0.1236
			GER-GMMG	0.47	511	156	116	244	151	1.25 (1.0-1.56)	0.0457
			US-UAMS	0.48	703	331	157	356	190	1.22 (1.05-1.43)	0.0106
			**Combined**								7.17×10^{-6}
											P_{het}=0.96, I^2=0%
**rs1974807(19q13.11)**	**33713058**	**C**					**CC**	**AC**	**AA**		
			UK-My9	0.19	1146	503	49	348	749	1.18 (1.02-1.37)	0.0244
			UK-My11	0.22	861	197	35	315	511	1.35 (1.07-1.7)	0.0107
			GER-GMMG	0.21	500	154	23	166	311	1.24 (0.93-1.64)	0.1384
			US-UAMS	0.21	693	326	28	238	427	1.28 (1.07-1.53)	0.0083
			**Combined**								8.88×10^{-6}
											P_{het}=0.8, I^2=0%
**rs2906053 (5q31.3)**	**141594215**	**C**					**CC**	**CT**	**TT**		
			UK-My9	0.11	1153	504	13	232	908	1.43 (1.19-1.72)	0.0001
			UK-My11	0.11	870	201	16	151	703	1.46 (1.1-1.92)	0.0077
			GER-GMMG	0.11	503	155	4	106	393	1.12 (0.8-1.58)	0.5081
			US-UAMS	0.11	672	316	9	131	532	1.18 (0.92-1.51)	0.2014
			**Combined**								4.93×10^{-6}
											P_{het}=0.41, I^2=0%
**rs4682170 (3q13.31)**	**114851633**	**A**					**AA**	**AC**	**CC**		
			UK-My9	0.15	1142	502	23	306	813	1.33 (1.12-1.58)	0.0013
			UK-My11	0.15	850	197	21	206	623	1.29 (0.99-1.69)	0.0579
			GER-GMMG	0.15	496	150	7	133	356	1.05 (0.77-1.43)	0.7765
			US-UAMS	0.13	686	319	16	151	519	1.47 (1.19-1.81)	0.0003
			**Combined**								9.8×10^{-7}
											P_{het}=0.36, I^2=7%
**rs57942319 (18q21.32)**	**56685448**	**A**					**AA**	**AG**	**GG**		
			UK-My9	0.08	1163	510	8	171	984	1.35 (1.1-1.67)	0.0050
			UK-My11	0.08	873	200	8	126	739	1.37 (1-1.88)	0.0493
			GER-GMMG	0.10	509	156	7	83	419	1.26 (0.87-1.81)	0.2209
			US-UAMS	0.08	700	331	4	105	591	1.5 (1.15-1.95)	0.0026
			**Combined**								$3.22. \times 10^{-6}$
											P_{het}=0.88, I^2=0%
**rs61070260 (2q22.1)**	**141603995**	**A**					**AA**	**AG**	**GG**		
			UK-My9	0.06	1132	500	3	120	1009	1.38 (1.06-1.79)	0.0153
			UK-My11	0.06	860	200	1	106	753	1.3 (0.89-1.9)	0.1721
			GER-GMMG	0.07	499	152	2	65	432	1.52 (1.05-2.21)	0.0279
			US-UAMS	0.07	685	324	2	91	592	1.52 (1.14-2.04)	0.0048
			**Combined**								8.05×10^{-6}
											P_{het}=0.9, I^2=0%

[a]Risk allele frequency (RAF). [b]Hazards ratio. [c]95% Confidence Interval.

Supplementary Table 2: Summary results for SNPs suggestive associations with MM-OS. *P*-values assessed by a cox proportional hazards model. Individual cohort results were combined by fixed odds meta-analysis.

MM OS associations	Region			6q25.1		1q23.3	19q13.11		5q31.3	3q13.31		18q21.32	2q22.1
	**SNP**			**rs12374648**		**rs1934908**	**rs1974807**		**rs2906053**	**rs4682170**		**rs57942319**	**rs61070260**
	**CHR**			6		1	19		5	3		18	2
	**BP**			151529369		161680868	33713058		141594215	114851633		56685448	141603995
	**A1**			G		C	C		C	A		A	A
	**A2**			A		T	A		T	C		G	G
	P			4.69×10^{-9}		7.17×10^{-6}	8.88×10^{-6}		4.93×10^{-6}	9.80×10^{-7}		3.22×10^{-6}	8.05×10^{-6}
	**Proximal genes**			MTHFD1L	AKAP12	FCRLA	LRP3	SLC7A10	NDFIP1	ZBTB20	SEMA5A	ZNF532	LRP1B
	**Typed SNP in LD**			rs9478986		rs1934908	rs8106493		rs2961694	rs6793973		rs8093617	rs16844912
		**Tissue type**	**Study size**										
**cis-eQTL**	**GTeX portal**	Mutiple	1,421	NS	NS	NS	NS	NS	NS	NS	NS	NS	NS
	**Blood eQTLs**	Whole blood	5,133	NA	NS	NS	NS	NS	NS	NS	NS	NS	NS
	**MuTHER - LCLs**	LCLs	850	NA	0.34	**1.12×10^{-7}**	0.72	0.17	0.55	NA	NA	NA	0.82
	**MuTHER - Adipose**	Adipose	850	NA	0.23	0.52	**0.063**	**3.06×10^{-9}**	0.32	NA	NA	NA	0.34
	**MuTHER - Skin**	Skin	850	NA	0.29	**3.12×10^{-6}**	0.943	0.17	0.41	NA	NA	NA	0.68
	**SCAN**	LCLs	176	NS	NS	NS	NS	NS	NS	NS	**7.0×10^{-5}**	NS	NS
**sQTL**	**Framingham Heart Study**	Whole blood	5,257	NA	NA	**1.24×10^{-46}**	NA	NA	NA	NA	NA	NA	NA
**Regulatory motifs**	**HaploReg v3**			Bound by TCF4		11 altered motifs	adipose enhancer, altered LUN-1, SETDB1		12 altered motifs	6 altered motifs		Bound by MAFK	4 altered motifs
	**Regulomedb**			TF binding, DNase peak		NS	TF binding		NS	NS		TF binding	NA

Supplementary Table 3: MM-OS SNPs in eQTL, mQTL and regulatory motif databases. Databases included in Table are: GTeX[1], Blood eQTL[2], MuTHER studies[3], Framingham heart study[4], SCAN[5], HaploReg[6] and Regulomedb[7]. eQTLs are labelled Non-significant (NS), where the eQTL value was not reported by database due to the weakness of the association. eQTLs are labelled Not applicable (NA) where expression on the gene was not present in the given cell type.

SNP	CHR	Base pair	A1	A2	N	*P*	*P(R)*	HR[a]	HR(R)	Q[b]	I^2 [c]
rs12374648	6q25.1	151529369	G	A	4	0.0001149	0.09487	1.1716	1.1393	0.0208	69.25
rs1934908	1q23.3	161680868	C	T	4	0.0001936	0.0006601	1.1258	1.1272	0.3185	14.71
rs1974807	19q13.11	33713058	C	A	4	0.003772	0.003772	1.1221	1.1221	0.8163	0
rs2906053	5q31.3	141594215	C	T	4	0.01526	0.01526	1.133	1.133	0.7085	0
rs4682170	3q13.31	114851633	A	C	4	0.1308	0.1308	1.0722	1.0722	0.7185	0
rs57942319	18q21.32	56685448	A	G	4	0.004558	0.004558	1.1721	1.1721	0.6818	0
rs61070260	2q22.1	141603995	A	G	4	0.02035	0.02035	1.1675	1.1675	0.5062	0

[a]Hazards ratio, [b]*p*-value for Cochrane's Q statistic, [c]Heterogeneity index

Supplementary Table 4: Relationship between suggestive associations with MM-OS SNPs and progression free survival. *P*-values assessed by a cox proportional hazards model. Individual cohort results were combined by fixed odds meta-analysis.

						Meta			UK-My9			UK-My11			GER-GMMG			US-UAMS		
SNP	CHR	Nearest gene	Base pair	A1	A2	*P*	HR	I^2	MAF	*P*	HR (95%CI)	MAF	*P*	HR (95%CI)	MAF	*P*	HR (95%CI)	MAF	*P*	HR (95%CI)
rs12622528*	2	*ST6GAL2*	107649440	T	C	0.402	1.04	5	0.33	0.075	1.13 (0.99-1.29)	0.33	0.734	1.04 (0.84-1.29)	0.34	0.827	0.97 (0.77-1.23)	0.35	0.459	0.94 (0.8-1.11)
rs6746082	2	*DNTB*	25659244	C	A	0.463	1.04	47	0.18	0.166	0.89 (0.75-1.05)	0.18	0.199	1.18 (0.92-1.53)	0.17	0.289	1.17 (0.88-1.55)	0.18	0.212	1.13 (0.93-1.38)
rs1052501	3	*ULK4*	41925398	C	T	0.835	0.99	0	0.20	0.847	1.02 (0.87-1.19)	0.20	0.675	0.95 (0.74-1.22)	0.19	0.183	0.81 (0.59-1.11)	0.19	0.610	1.05 (0.87-1.27)
rs10936599	3	*TERC*	169492101	T	C	0.601	1.03	0	0.20	0.775	1.02 (0.88-1.19)	0.20	0.479	1.09 (0.86-1.39)	0.20	0.691	1.06 (0.8-1.39)	0.23	0.858	0.98 (0.81-1.19)
rs56219066	5	*ELL2*	95242931	C	T	0.603	1.03	0	0.24	0.499	1.05 (0.91-1.21)	0.21	0.978	1.00 (0.79-1.26)	0.22	0.542	1.08 (0.82-1.43)	0.23	0.797	0.98 (0.81-1.17)
rs2285803	6	*POU5F1*	31107258	T	C	0.169	1.06	0	0.32	0.209	1.09 (0.95-1.25)	0.31	0.242	1.13 (0.92-1.39)	0.36	0.269	1.14 (0.9-1.45)	0.32	0.546	0.95 (0.81-1.12)
rs4487645	7	*DNAH11*	21938240	A	C	0.895	0.99	0	0.28	0.394	0.94 (0.82-1.08)	0.31	0.376	1.1 (0.89-1.34)	0.25	0.774	1.04 (0.81-1.32)	0.29	0.897	0.99 (0.83-1.17)
rs4273077	17	*TNFRSF13B*	16849139	G	A	0.742	0.98	64	0.12	0.788	1.03 (0.85-1.23)	0.12	0.037	0.68 (0.47-0.98)	0.15	0.085	1.29 (0.97-1.73)	0.11	0.287	0.87 (0.68-1.12)
rs877529	22	*CBX7*	39542292	A	G	0.519	0.97	0	0.49	0.441	1.05 (0.93-1.19)	0.50	0.606	1.05 (0.86-1.28)	0.44	0.407	1.1 (0.88-1.39)	0.49	0.676	1.03 (0.89-1.21)

[a]Minor allele frequency (MAF). [b]Hazards ratio. [c]95% Confidence Interval, * R2=1, proxy for rs12614346

Supplementary Table 5: Relationship between susceptibility SNPs for MM and MM-OS. *P*-values assessed by a cox proportional hazards model. Individual cohort results were combined by fixed odds meta-analysis.

Trend

	RAF[a]	HR[b]	95% CI[c]	*P*	
UK-My9	0.062	0.92	0.71-1.19	0.539	
UK-My11	0.048	1.47	0.97-2.26	0.069	
GER-GMMG	0.059	1.03	0.67-1.60	0.878	
US-UAMS	0.057	0.86	0.62-1.19	0.37	
**Combined**		0.99	0.84 -1.19	0.921	I^2=33%

AA vs AT + TT

	HR	95% CI	*P*	
UK-My9	0.97	0.85-1.10	0.642	
UK-My11	1.23	0.99-1.52	0.062	
GER-GMMG	1.06	0.83-1.36	0.635	
US-UAMS	0.94	0.80-1.11	0.486	
**Combined**	1.01	0.93-1.10	0.794	I^2=31%

[a]Risk allele frequency (RAF). [b]Hazards ratio. [c]95% Confidence Interval

Supplementary Table 6: Association statistics for the SNP at locus 16p13 rs72773978. *P*-values assessed by a cox proportional hazards model. Individual cohort results were combined by fixed odds meta-analysis.

Supplementary References

1. Consortium, G.T. Human genomics. The Genotype-Tissue Expression (GTEx) pilot analysis: multitissue gene regulation in humans. *Science* **348**, 648-60 (2015).
2. Westra, H.J. *et al.* Systematic identification of trans eQTLs as putative drivers of known disease associations. *Nat Genet* **45**, 1238-43 (2013).
3. Nica, A.C. *et al.* The architecture of gene regulatory variation across multiple human tissues: the MuTHER study. *PLoS Genet* **7**, e1002003 (2011).
4. Zhang, X. *et al.* Identification of common genetic variants controlling transcript isoform variation in human whole blood. *Nat Genet* **47**, 345-52 (2015).
5. Gamazon, E.R. *et al.* SCAN: SNP and copy number annotation. *Bioinformatics* **26**, 259-62 (2010).
6. Ward, L.D. & Kellis, M. HaploReg: a resource for exploring chromatin states, conservation, and regulatory motif alterations within sets of genetically linked variants. *Nucleic Acids Res* **40**, D930-4 (2012).
7. Boyle, A.P. *et al.* Annotation of functional variation in personal genomes using RegulomeDB. *Genome Res* **22**, 1790-7 (2012).
